# Hypoxia delays steroid-induced developmental maturation in *Drosophila* by suppressing EGF signaling

**DOI:** 10.1371/journal.pgen.1011232

**Published:** 2024-04-26

**Authors:** Michael J. Turingan, Tan Li, Jenna Wright, Abhishek Sharma, Kate Ding, Shahoon Khan, Byoungchun Lee, Savraj S. Grewal

**Affiliations:** Clark H Smith Brain Tumour Centre, Arnie Charbonneau Cancer Institute, Alberta Children’s Hospital Research Institute, and Department of Biochemistry and Molecular Biology Calgary, University of Calgary, Alberta, Canada; University of Helsinki: Helsingin Yliopisto, FINLAND

## Abstract

Animals often grow and develop in unpredictable environments where factors like food availability, temperature, and oxygen levels can fluctuate dramatically. To ensure proper sexual maturation into adulthood, juvenile animals need to adapt their growth and developmental rates to these fluctuating environmental conditions. Failure to do so can result in impaired maturation and incorrect body size. Here we describe a mechanism by which *Drosophila* larvae adapt their development in low oxygen (hypoxia). During normal development, larvae grow and increase in mass until they reach critical weight (CW), after which point a neuroendocrine circuit triggers the production of the steroid hormone ecdysone from the prothoracic gland (PG), which promotes maturation to the pupal stage. However, when raised in hypoxia (5% oxygen), larvae slow their growth and delay their maturation to the pupal stage. We find that, although hypoxia delays the attainment of CW, the maturation delay occurs mainly because of hypoxia acting late in development to suppress ecdysone production. This suppression operates through a distinct mechanism from nutrient deprivation, occurs independently of HIF-1 alpha and does not involve dilp8 or modulation of Ptth, the main neuropeptide that initiates ecdysone production in the PG. Instead, we find that hypoxia lowers the expression of the EGF ligand, spitz, and that the delay in maturation occurs due to reduced EGFR/ERK signaling in the PG. Our study sheds light on how animals can adjust their development rate in response to changing oxygen levels in their environment. Given that hypoxia is a feature of both normal physiology and many diseases, our findings have important implications for understanding how low oxygen levels may impact animal development in both normal and pathological situations.

## Author summary

Animals face various environmental challenges during their growth, such as changes in food availability, temperature, and oxygen levels. Inability to adapt to these changes can cause developmental problems. Here we explore how Drosophila larvae adapt to low oxygen conditions (known as hypoxia). Typically, larvae grow until they achieve a critical weight, which triggers the release of a hormone called ecdysone that initiates their transformation into the pupal stage. Under hypoxic conditions (5% oxygen), we observed that the larvae’s growth slows down and their maturation is delayed. Our research indicates that this delay is mainly due to a reduction in ecdysone production towards the end of their development caused by hypoxia. We found that these effects of hypoxia occur due to decreases the expression of the epidermal growth factor (EGF) ligand, spitz, which in turn reduces EGFR/ERK signaling in the prothoracic gland, the gland responsible for producing ecdysone. These findings help explain how animals regulate their development in response to changes in oxygen levels and have broader implications for understanding conditions, both normal and pathological, that are characterized by low oxygen environments.

## Introduction

Animals often grow and develop in unpredictable environments. Fluctuations in nutrient availability, temperature, or oxygen and exposure to toxins and pathogens can create conditions of environmental stress. Animals must, therefore, be able to assess these conditions and, in turn, adapt their physiology and growth rate to ensure proper development and attainment of functional organ and body size [1–7]. Any defects in these adaptations can impair fitness. Understanding how environmental signals control animal development is, therefore, an important question in biology.

In animals that exhibit determinate growth, increases in body size occur during the juvenile stage of development before they mature to an adult stage, and further growth stops. Final body size is thus determined by the duration of the juvenile growth stage and the timing of maturation. *Drosophila* has provided a tractable and valuable model system to understand how environmental conditions can control the timing of juvenile maturation [8,9]. In *Drosophila*, body growth occurs during the larval (juvenile) period where animals increase ~200-fold in mass over a 4–5 day period before undergoing maturation to the pupal stage and metamorphosis into adults [10,11]. Since adults do not grow, the timing of larval maturation is a key determinant of final body size. Larval maturation is controlled by steroid hormone ecdysone which is produced and secreted from the prothoracic gland (PG). Small pulses of ecdysone secretion time the moults from one larval stage to the next before a final, large pulse of ecdysone secretion acts on larval tissues to trigger the irreversible maturation from the larval to pupal stage [8]. This final maturation-inducing pulse of ecdysone is controlled by a brain-to-PG neuroendocrine signaling circuit. Two subsets of neurons in the brain control the production of this ecdysone pulse–a bilateral pair of Ptth neurons and a bilateral subset of serotonergic neurons. These neurons directly innervate the PG and, when activated, they stimulate the expression of ecdysone biosynthetic genes and production of ecdysone in the PG [12–14]. In addition, autocrine Epidermal Growth Factor Receptor (EGFR) signaling is a major regulator of ecdysone production in the PG. At the end of larval development, the expression of two EGFR ligands—Vein and Spitz–increases in the PG. Vein and Spitz can then act in an autocrine manner to stimulate EGFR and activate the conserved Ras /ERK signaling pathway in PG cells to upregulate the expression of ecdysone biosynthesis genes and promote ecdysone-dependent larval maturation [15].

An array of secreted peptides, cytokines and growth factors can signal from other cells and tissues to act on the brain-PG network to control the timing of ecdysone production and maturation [8,16–19]. These signals allow larvae to integrate both intrinsic signals and environmental cues to ensure that larval maturation occurs only when the animals are competent to undergo successful metamorphosis during the pupal stage to produce viable, properly sized adults. For example, damage to the larval imaginal discs or disruption of their growth leads to a delay in larval maturation to allow time for the injured or slow-growing tissues to regenerate and grow to their correct size [20]. This developmental delay is mediated by the relaxin/insulin-like peptide dilp8 which is produced from damaged discs and signals to subset of neurons in the brain that express lgr3, the dilp8 receptor [21–28]. These lgr3 neurons directly connect to the ptth neurons and are thought to inhibit their activity, thus suppressing the stimulation of ecdysone production. Thus, this dilp8-lgr3 signaling network functions as a developmental checkpoint to ensure that larvae initiate maturation to the pupal stage only when the imaginal discs–which give rise to most of the adult structures–are properly formed.

One of the most well-understood environmental factors influencing larval maturation is nutrient availability [17,29]. For transition to the pupal stage to occur, larvae need to attain an appropriate mass and level of nutrient stores—defined as critical weight (CW)—to support the energetically costly process of metamorphosis during the pupal stage [17]. Once larvae pass this developmental growth checkpoint, they initiate the process of ecdysone production in the PG and maturation to the pupal stage even if they are subsequently starved of nutrients. The conserved insulin/PI3K and TOR kinase signaling pathways are the main nutrient sensing and signaling pathways in larvae [18,30–32]. In rich nutrient conditions, upregulation of insulin/PI3K and TOR signaling stimulates the cell and tissue growth that drives accumulation of body mass during larval development, while low nutrient conditions lead to reduced insulin and TOR signaling and slow body growth [18,30]. Several studies have shown that stimulation of insulin/PI3K and TOR signaling in the PG provides a mechanism to couple nutrient-dependent growth and nutritional status to CW assessment and the initiation of larval maturation [33–39]. For example, as larvae grow and approach CW, nutrient stimulation of TOR kinase signaling in the PG can initiate the process of ecdysone production and larval maturation through regulating activity of the transcription factor, Snail, promoting PG cell endoreplication and modulating autophagy [36,37,40]. Insulin/PI3K signaling in the PG can also control nutrient-dependent ecdysone production through regulation of the transcription factor, FOXO [34]. Prior to CW attainment, FOXO inhibits expression of ecdysone synthesis genes. But as larvae feed and grow and reach CW, activation of insulin/PI3K signaling in PG cells by systemically circulating insulin-like peptides leads to inhibition of FOXO thus allowing expression of ecdysone biosynthesis and the onset of maturation [34]. Once larvae pass the CW checkpoint, they can progress to the pupal stage even if they are nutrient starved. In this case, larvae rely on an endocrine tissue called the corpora cardiaca that lies adjacent to the PG to release insulin-like peptides that act on the PG to promote the synthesis of ecdysone required for maturation [39]. In addition to these insulin/PI3K and TOR dependent mechanisms controlling PG ecdysone production, the serotonergic neurons that innervate the PG have been shown to link nutrient availability to the production of ecdysone in the PG [14], while Ptth has been shown to be important for allowing larvae to adapt the timing of their maturation to poor nutrient and crowded growth conditions [12]. Thus, larvae can utilize multiple nutrient sensing and signaling mechanisms to ensure that nutrient-dependent growth is robustly and accurately coupled to the onset of larval maturation.

Oxygen availability is another key environmental cue that can modulate animal development [41–44]. *Drosophila* larvae grow and develop by burrowing into fermenting food rich in microorganisms, an environment that is likely characterized by low oxygen or hypoxia [4,45]. Hence, they have evolved mechanisms to be able to adapt their development to conditions of hypoxia. When grown in moderate hypoxia (5–10% oxygen) larvae slow their growth rate and delay their maturation leading to smaller sized adults [4,5,46–54]. Studies have described how the reduction in growth rate occurs through reduced insulin and TOR kinase signaling [47–50,55]. However, the mechanisms by which hypoxia delays the timing of larval maturation remain to be determined. We explore this issue in this study.

## Results

### Hypoxia slows development and delays larval maturation

**Fig 1:**
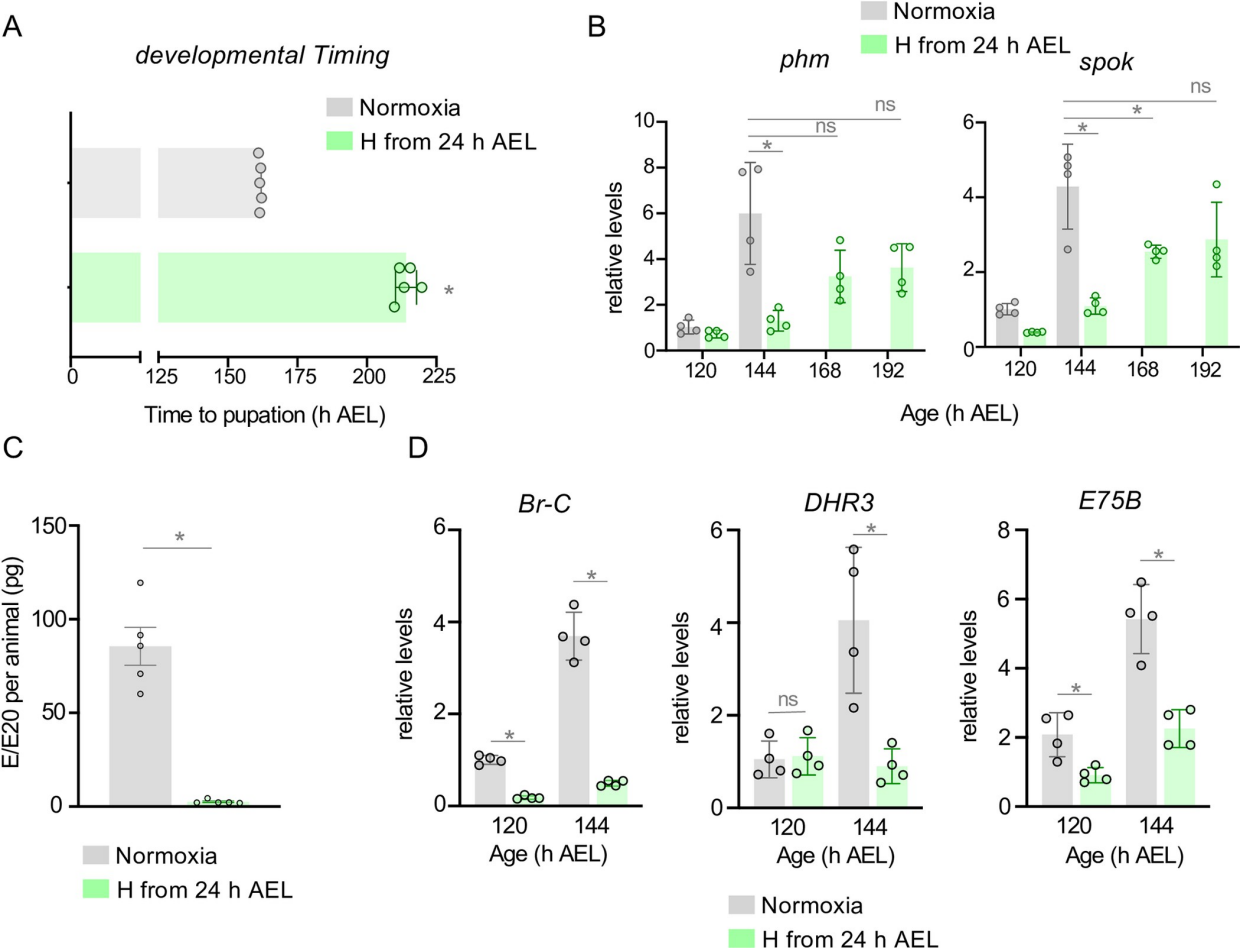
Hypoxia delays ecdysone production and larval maturation. **(A)** Mean time to pupation of *w*^*1118*^ larvae reared in ambient oxygen or 5% O_2_ (from hatching), in hours after egg laying (h AEL). Each data point represents the mean time to pupation of a vial of 30 larvae. n (# of vials) ≥ 3 per condition. Bars represent mean +/- SEM with individual data points plotted as symbols. **(B)** Relative mRNA levels (normalized to Rpl32) of ecdysone biosynthetic genes *phantom* (*phm*) and *spookier* (*spok*) from whole-larvae qRT-PCR of *w*^*1118*^ larvae reared in ambient oxygen or in 5% O2 from hatching (24 h AEL). n (# of independent samples) ≥ 3 per condition. Bars represent mean +/- SEM with individual data points plotted as symbols. **(C)** Hemolymph titers of E/E20 from *w*^*1118*^ larvae reared in ambient air or in 5% O_2_ from hatching (24 h AEL). n (# of independent samples) = 5 groups of larvae per condition. Bars represent mean +/- SEM with individual data points plotted as symbols. **(D)** Relative mRNA levels of ecdysone target genes, *Br-C*, *DHR3*, and *E75B* from whole-larvae qRT-PCR of *w*^*1118*^ larvae reared in ambient oxygen or in 5% O_2_ from hatching (24 h AEL). n (# of independent samples) = 4 groups of larvae per condition. Bars represent mean +/- SEM with individual data points plotted as symbols. * denotes p < 0.05. Also see S1 Fig.

We raised larvae in normoxia or hypoxia (5% oxygen) from hatching and measured the time to pupariation. We saw that the hypoxia exposed larvae showed an approximate two-day delay in development (Fig 1A). As previously described [49, 50], this was accompanied by a reduction in final body size but without any reduction in larval viability (S1A and S1B Fig). These results show that hypoxia exposed larvae have reduced body growth and a delay in development to the pupal stage. Larval maturation is regulated by a neuroendocrine circuit that produces a pulse of ecdysone from the prothoracic gland (PG) which triggers the larval to pupal transition [8]. To measure this ecdysone pulse, we used qPCR to quantify mRNA levels of two Halloween genes, *phm* and *spok*, that are involved in ecdysone biosynthesis in the PG. We found that, in normoxia, the expression of these genes peaked at 144 hr AEL (after egg laying), prior to the larval to pupal transition (Fig 1B). However, in the hypoxia-exposed larvae the increase in expression of both *phm* and *spok* were delayed and blunted compared to their normoxic counterparts (Fig 1B). We also found that hypoxia exposure strongly suppressed hemolymph ecdysone titers in late third instar larvae (Fig 1C). Finally, we saw a hypoxia-mediated decrease in the whole-body expression of the ecdysone downstream target genes, *Br-C*, *DHR3* and *E75B* consistent with reduced ecdysone signaling (Fig 1D). These results indicate that the hypoxia-induced delay in larval maturation is associated with both a delay and reduction in the final larval ecdysone pulse.

### Hypoxia acts post critical weight to suppress ecdysone signaling

**Fig 2:**
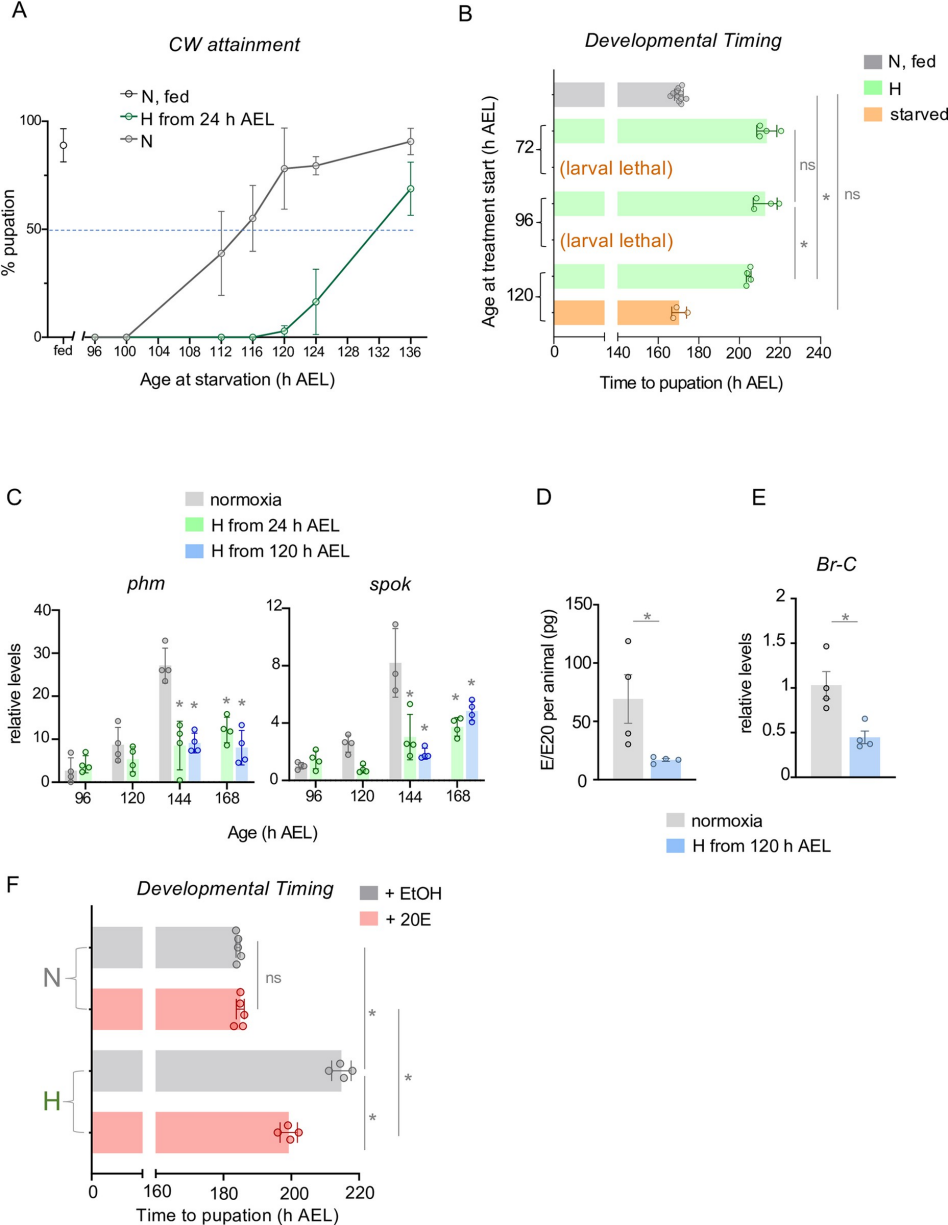
Hypoxia delays ecdysone production and larval maturation independent of critical weight attainment. **(A)** Determination of age at Critical weight (CW) attainment for animals reared in ambient or 5% O_2_ (Hypoxia, ‘H’) conditions. The age at CW attainment (in h AEL) was defined as the age which yielded 50% pupation upon starvation. Each data point represents the mean of three vials of 30 larvae. n (# of vials of 30) ≥ 3 per condition. Data presented as mean +/- SEM. **(B)** Mean time to pupation of *w*^*1118*^ larvae reared in ambient oxygen or 5% O_2_ (from hatching), in hours after egg laying (h AEL). Each data point represents a vial of 30 larvae. n (# of vials) ≥ 3 per condition. Data presented as mean +/- SEM. **(C)** Relative mRNA levels (normalized to Rpl32) of ecdysone biosynthetic genes *phantom* (*phm*) and *spookier* (*spok*) from whole-larvae qRT-PCR of *w*^*1118*^ larvae reared in ambient oxygen or in 5% O2 from hatching (24 h AEL). n (# of independent samples) ≥ 3 per condition. **(D)** Hemolymph titers of E/E20 from *w*^*1118*^ larvae reared in ambient air or reared in ambient air and then exposed to hypoxia (5% O_2_) post CW (at 120h AEL). n (# of independent samples) = 4 groups of larvae per condition. Bars represent mean +/- SEM with individual data points plotted as symbols. **(E)** Relative mRNA levels of the ecdysone target gene, *Br-C*, from whole-larvae qRT-PCR of *w*^*1118*^ larvae reared in ambient air or reared in ambient air and then exposed to hypoxia (5% O_2_) post CW (at 120h AEL). n (# of independent samples) = 4 groups of larvae per condition. Bars represent mean +/- SEM with individual data points plotted as symbols. * denotes p < 0.05. **(F)** Developmental timing of *w*^*1118*^ larvae fed either 20-hydroxyecdysone (20E) or ethanol (EtOH) vehicle, reared in either ambient oxygen or 5% oxygen from 120 h AEL (*i*.*e*., post-CW). n (# of vials of 30) ≥ 3 per condition. * denotes p < 0.05; ns denotes non-significant. Also see S2 and S3 Figs.

A key checkpoint for the initiation of larval maturation is the attainment of critical weight (CW), a point in larval development where animals have achieved sufficient mass and nutrient stores to complete development to viable adults without any further nutrients [9, 29]. CW is determined, in large part, by an animal’s growth rate, which in *Drosophila* larvae is controlled by the conserved insulin and TOR kinase signaling pathways [17]. Given that hypoxia exposure can suppress larval growth by inhibiting these pathways [47–50, 55], it is possible that the delay in maturation merely reflects a hypoxia-induced delay in attainment of CW. To address this, we raised larvae from hatching in either normoxia or hypoxia and then at defined times in development we switched them to starvation conditions and monitored their subsequent development in normoxia. CW can be assessed by determining the time point in development at which >50% larvae can pupariate when subsequently starved. We found that in normoxia this point was reached at ~114 hrs AEL, whereas in hypoxia-exposed animals, this point was at ~132hrs AEL, indicating that hypoxia exposure does delay CW (Fig 2A). We then examined the effects of hypoxia exposure introduced either before or after CW attainment. We raised larvae from hatching in normoxia and then at 72hrs, 96 hrs or 120 hrs AEL we switched larvae either to hypoxia or starvation conditions and then monitored their subsequent development. As expected, we found that larvae starved at 72 and 96hrs (pre-CW) arrested their development and failed to pupate while larvae starved at 120 hrs (post CW) completed larval development and pupated at the same time as control (normoxic, fed) larvae but at a significantly smaller final size (Figs 2B, S2A and S2B). However, we found that larvae exposed to hypoxia at all three time points were able to pupate but showed a delay to pupariation (Figs 2B and S2A). And, interestingly, larvae switched to hypoxia post CW (120hrs AEL) showed a delay in development (~34hrs) that was only slightly shorter than that seen with pre-CW larvae switched to hypoxia at 72hrs AEL (43 hr delay) or 96hrs AEL (42 hrs delay) (Fig 2B). We also found that animals switched to hypoxia exposure at each of the three timepoints had significantly reduced pupal sizes compared to normoxic controls, but that animals exposed to hypoxia post-CW were significantly larger than those exposed to hypoxia pre-CW (S2B Fig). Together these results indicate that, although hypoxia early in larval development can slow growth and delay CW attainment, hypoxia can also act specifically at the late larval stage to suppress the mechanism(s) that promote maturation. Consistent with this we saw that larvae exposed to hypoxia post CW (120hrs AEL) showed a similar suppression of whole-body *phm* and *spok* mRNA levels as larvae exposed to hypoxia from hatching (Fig 2C), suggesting that the hypoxia-induced decrease in ecdysone biosynthesis is largely due to an effect of low oxygen in late larval development. This post-CW hypoxia suppression of *phm* and *spok* mRNA levels was also seen when we measured mRNA levels in isolated ring glands (S2C Fig). We also found that larvae exposed to hypoxia post CW (120hrs AEL) showed a reduction in hemolymph ecdysone titres (Fig 2D) and decreased whole-body expression of the ecdysone target gene, *Br-C* (Fig 2E). Furthermore, we found that feeding larvae 20-hydroxyecdysone could partially but significantly reverse the delay in development seen in larvae that were switched to hypoxia post CW (at 120hrs AEL) and led to smaller sized animals compared to vehicle-fed hypoxic animals (Figs 2F and S2D). These results suggest that hypoxia can act on the mechanisms that control the timing of the late larval ecdysone pulse.

Our results also suggest that larvae may use different mechanisms to couple nutrient availability and oxygen levels to the control of maturation timing. Previous work has described how stimulation of TOR kinase activity and inhibition of FOXO in the PG are two key mechanisms that couple nutritional status to the attainment of CW and larval maturation, and that genetic activation of TOR signaling or suppression of FOXO activity in the PG can reverse the delay in CW attainment and larval maturation seen in low nutrient conditions [34,35]. However, we found that neither overexpression of the TOR activator, Rheb, or knockdown of FOXO in the PG could reverse the hypoxia-mediated delay in larval development to the pupal stage (S3A and S3C Fig). Rheb overexpression in the PG also had no significant effect on final size in either normoxia- or hypoxia-exposed animals (S3B Fig). Importantly, we found that hypoxia had no effect on the expression of Gal4 from the two PG drivers (*P0206-Gal4* and *spok-Gal4)* that were used for these experiments as indicated by visualization of *UAS-GFP* (S3D Fig). These results provide further evidence that nutrient restriction and hypoxia may operate through different mechanisms to delay larval maturation.

### The hypoxia-induced maturation delay is independent of HIF1-alpha/sima function

**Fig 3:**
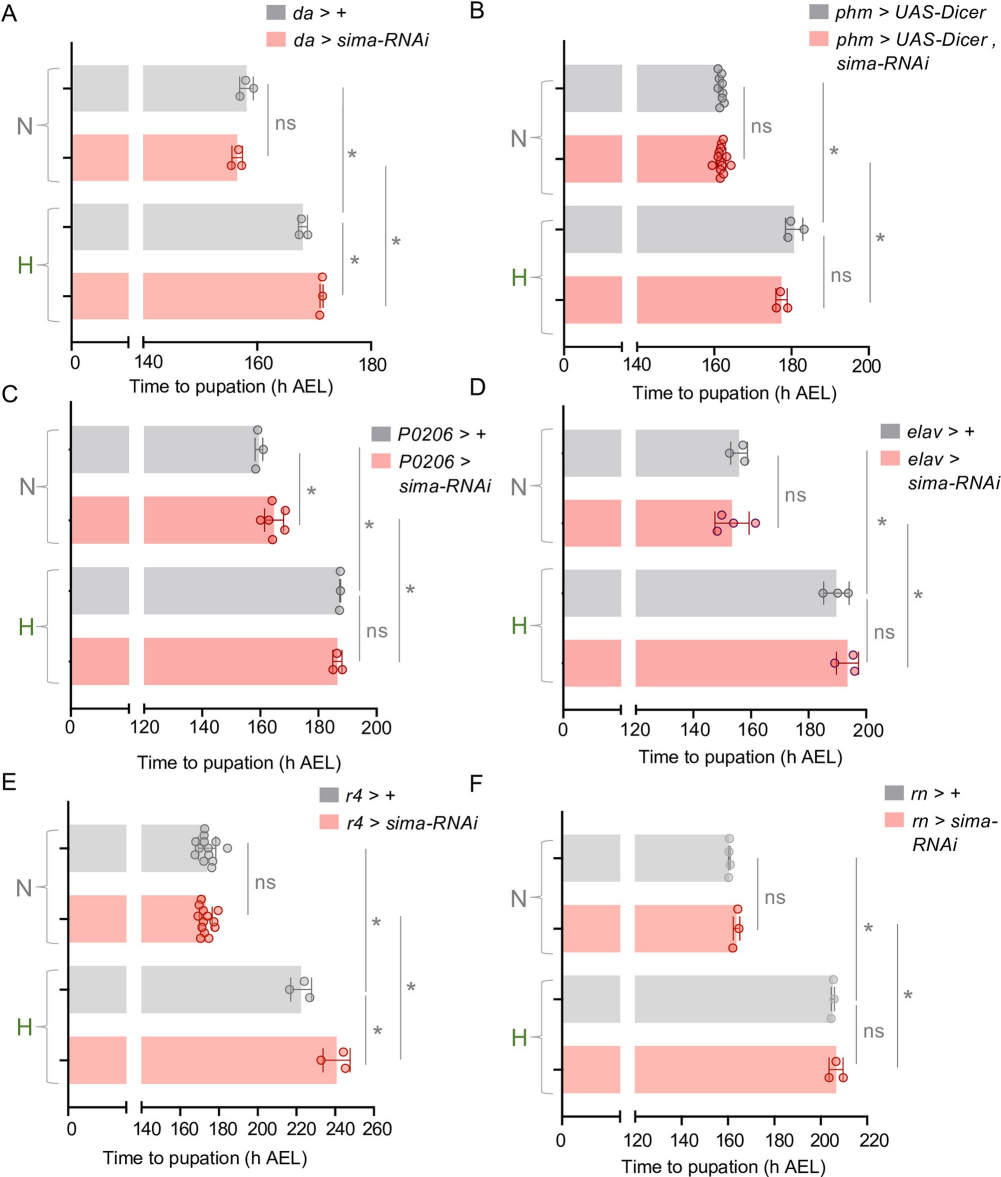
The hypoxia-induced developmental delay is independent of sima/HIF-1α. **(A-C)** Mean time to pupation of larvae of the indicated genotype reared in either normal oxygen conditions throughout development (‘N’) or shifted to 5% O_2_ at 120 h AEL (‘H’). Knockdown of *sima* was performed, **(A)** ubiquitously (*da-Gal4*), **(B)** in the prothoracic gland (*phm-GAL4*, *UAS-dicer*), **(C)** in the prothoracic gland/corpus allatum (*P0206-GAL4*), (**D)** pan-neuronally (*elav-GAL4*), **(E)** in the fat body (*r4-Gal4)*, or **(F)** in imaginal discs (*rn-Gal4)*. n (# of vials of 30) ≥ 3 per condition. Bars represent mean +/- SEM with individual data points plotted as symbols. * denotes p < 0.05; ns denotes non-significant. Also see S4 Fig.

The conserved hypoxia-inducible factor 1 alpha (HIF-1 alpha), known as *sima* in *Drosophila*, is perhaps the best characterized mediator of hypoxia responses in metazoans [56]. In normoxia, HIF-1 alpha protein is continually synthesized but then rapidly degraded. However, upon hypoxia exposure this degradation is blocked, HIF-1 alpha protein accumulates, and it can then translocate to the nucleus and control the expression of genes needed for hypoxic adaptive responses [56]. Previous studies have shown that *sima* can modulate growth and survival during periods of hypoxia in flies [49, 57–60]. We therefore explored whether *sima* might mediate the maturation delay seen in hypoxia. We began by examining the effects of the ubiquitous knockdown of *sima* using the *da-Gal4* driver. We previously showed that this approach leads to a strong knockdown of *sima* mRNA and can mimic the decreased lethality seen in *sima* mutants when larvae are exposed to hypoxia from hatching [50]. We also saw that ubiquitous *sima* knockdown leads to strong blunting of the hypoxia-mediated induction of the sima target gene, *fatiga* (S4A Fig). To examine a potential role for sima in mediating the effects of hypoxia on maturation, we compared developmental timing in control (*da > +)* vs ubiquitous *sima* knockdown (*da > sima RNAi)* larvae that were maintained in normoxia or exposed to hypoxia post CW. We found that sima knockdown had no effect in normoxia-exposed animals and led to a small, but significant exacerbation of the hypoxia-mediated delay in maturation (Fig 3A). We also examined the effects of tissue-specific *sima* knockdown. Given the central role for the brain-PG neuroendocrine circuit in controlling ecdysone production and larval maturation, we tested the effects of RNAi-mediated knockdown of *sima* in the PG. We found that RNAi-knockdown of *sima* using the PG driver, *phm-Gal4*, did not lead to a reversal in the hypoxia-induced delay in larval maturation (Fig 3B) and did not affect final pupal size in hypoxia (S4B Fig). Knockdown of *sima* with another PG driver, *P0206-GAL4*, led to a modest delay in development in normoxia but did not reverse the hypoxia-induced delay in the larval-to-pupal transition (Fig 3C). We next examined a role for neuronal *sima* in the hypoxia-mediated delay in maturation. We used the *elav-Gal4* line to drive *UAS-sima RNAi* specifically in all post-mitotic neurons. We found that neuronal-specific knockdown of *sima* had no effect on the timing of larval maturation in animals grown in either normoxia or hypoxia (Fig 3D). Similar results were seen when we used a second independent *UAS-Sima RNAi* line (S4C Fig). Finally, we examined the role for sima in two peripheral tissues–imaginal discs and fat body—that have been shown to signal to the brain and/or PG to control ecdysone production and larval maturation. Using the fat body driver *r4-Gal4* we found that RNAi-mediated knockdown of *sima* had no effect on developmental timing in normoxia and led to a small but significant exacerbation of the hypoxia-induced delay in the time to pupariation (Fig 3E). We also saw that knockdown of sima using *rn-Gal4*, an imaginal disc driver, also had no effect on larval maturation in either normoxia- or hypoxia-exposed animals (Fig 3F). Taken together, these results suggest that the hypoxia-mediated delay in larval maturation does not rely on induction of HIF1-alpha/sima.

### The hypoxia-induced maturation delay is independent of dilp8 and Ptth/Torso signaling

**Fig 4:**
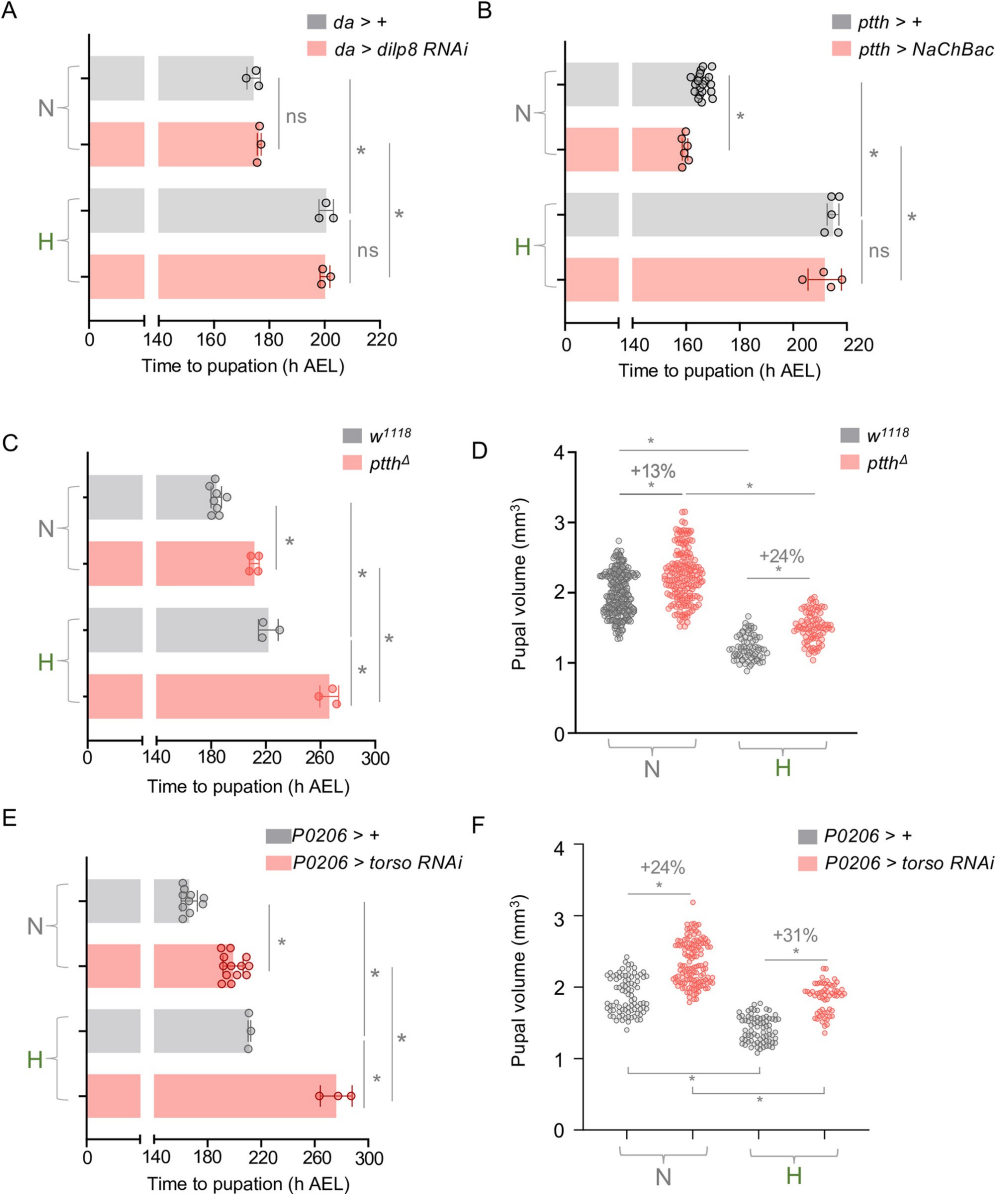
The hypoxia-induced maturation delay is independent of dilp8 and Ptth/Torso signaling. **(A)** Mean time to pupation of either control (*da > +*) or dilp8 knockdown (*da > dilp8* RNAi) larvae reared in either normal oxygen conditions throughout development (‘N’) or shifted to 5% O_2_ at 120 h AEL (‘H’). n (# of vials of 30 larvae) ≥ 3 per condition. Bars represent mean +/- SEM with individual data points plotted as symbols. **(B)** Mean time to pupation of either control larvae (*ptth > +*) or larvae expressing the bacterial sodium channel, NaChBac (*ptth > NaChBac*) reared in either normal oxygen conditions throughout development (‘N’) or shifted to 5% O_2_ at 120 h AEL (‘H’) n (# of vials of 30 larvae) ≥ 3 per condition. Bars represent mean +/- SEM with individual data points plotted as symbols. **(C)** Mean time to pupation of larvae, either *w*^*1118*^ or mutant for *ptth*, reared in either normal oxygen conditions throughout development (‘N’) or shifted to 5% O_2_ at 120 h AEL (‘H’) n (# of vials of 30 larvae) ≥ 3 per condition. Bars represent mean +/- SEM with individual data points plotted as symbols. **(D)** Pupal size of animals, either *w*^*1118*^ or mutant for *ptth*, reared in normoxia or hypoxia from 120 h AEL. Each data point represents body size measured for one animal. n (# of pupae) = 254 (N *w*^*1118*^), 172 (N *ptth*^*Δ*^), 70 (H *w*^*1118*^), 79 (H *ptth*^*Δ*^). **(E)** Mean time to pupation of larvae, either *P0206 >* + or *P0206 > torso RNAi*, reared in either normal oxygen conditions throughout development or shifted to 5% O_2_ at 120 h AEL. n (# of vials of 30 larvae) ≥ 3 per condition. Bars represent mean +/- SEM with individual data points plotted as symbols. **(F)** Pupal size of animals, either *P0206 >* + or *P0206 > torso RNAi*, reared in normoxia or hypoxia from 120 h AEL. Each data point represents body size measured for one animal. n (# of pupae) = 86 (N *P0206 >* +), 127 (N *P0206 > torso RNAi*), 75 (H *P0206 >* +), 59 (H *P0206 > torso RNAi*). * denotes p < 0.05. Also see S5 and S6 Figs.

The secreted factor dilp8 has emerged as a key regulator of larval developmental timing. When imaginal discs are damaged or experience disrupted growth, they induce dilp8 expression, which then signals from these tissues to the brain to suppress the Ptth-mediated induction of the maturation-inducing late larval ecdysone pulse [26]. We were therefore interested in exploring whether alterations in dilp8 and/or Ptth signaling could control developmental timing during hypoxia. Environmental stresses such as irradiation or ingestion of chemical mutagens have been shown to delay larval maturation due to damage-induced upregulation of imaginal disc dilp8 signaling [22,25]. Therefore, we first examined disc dilp8 expression using dilp8-GFP reporter line. We saw low levels of dilp8-GFP in the wing imaginal disc pouch, as has been previously reported. However, we found that hypoxia exposure had no effect on this dilp8 expression (S5A Fig). When we measured whole-body *dilp8* mRNA levels using qRT-PCR, we also saw that hypoxia exposure, either throughout the larval period or post-CW, had no effect on dilp8 mRNA levels (S5B Fig). Finally, we tested the effects of ubiquitous expression of a *UAS-dilp8 RNAi* transgene using the *da-Gal4* driver. Using two independent UAS-dilp8 RNAi lines, we found that whole-body dilp8 knockdown had no effect on hypoxia-induced delay in development (Figs 4A and S5C). These results suggest that hypoxia exposure does delay larval maturation by upregulating dilp8 signaling.

We next explored whether the hypoxia-induced delay in larval maturation might occur due to suppression of Ptth signaling. We first examined whether stimulation of the Ptth neurons would prevent the delayed development caused by hypoxia exposure. To do this we used the *ptth-Gal4* line to overexpress the NaChBac sodium channel which leads to increased neuronal excitation. We found that stimulating the Ptth neurons in this way accelerated larval maturation in normoxia (Fig 4B), consistent with upregulation of Ptth signaling to the PG and precocious induction of the maturation-inducing ecdysone pulse. However, we found that electrical stimulation of Ptth neurons had no effect on the hypoxia-induced delay in pupariation (Fig 4B). We next explored the effects of inhibiting Ptth signaling. We reasoned that if hypoxia functions by inhibiting Ptth signaling, then suppression of this pathway would not show any additive delay in development when combined with hypoxia exposure. We began by looking at *ptth* mutants. As described previously we found that, in normoxia, *ptth* null mutants showed a delay in pupariation and a 13% increase in final pupal size (Fig 4C and 4D) [12,13], consistent with a role for the Ptth signaling in the control of timing the late larval ecdysone pulse. However, we found that this delay in maturation in hypoxia-exposed larvae was further exacerbated with loss of *ptth* (Fig 4C) and hypoxia-exposed *ptth* mutants now showed a 24% increase in final pupal size (Fig 4D). We saw similar effects with another independent *ptth* mutant (S6A and S6B Fig). We also examined the effects of knockdown of the Ptth receptor, Torso, in the PG. We found that RNAi-mediated knockdown of *torso* in the PG of normoxic animals led to a delay in pupariation and a 24% increase in pupal size (Fig 4E and 4F). However, as with loss of Ptth, the delay in development seen in hypoxia exposed larvae was further exacerbated in animals with *torso* knockdown (Fig 4E) and *torso* knockdown animals now showed a 31% increase in pupal size (Fig 4F). Together, these additive effects of hypoxia and loss of Ptth/Torso signaling on larval maturation suggest that the effects of hypoxia on larval maturation are not explained by a suppression of Ptth/Torso signaling.

### Hypoxia induces a delay in maturation by suppressing spitz-EGFR signaling in the PG

**Fig 5:**
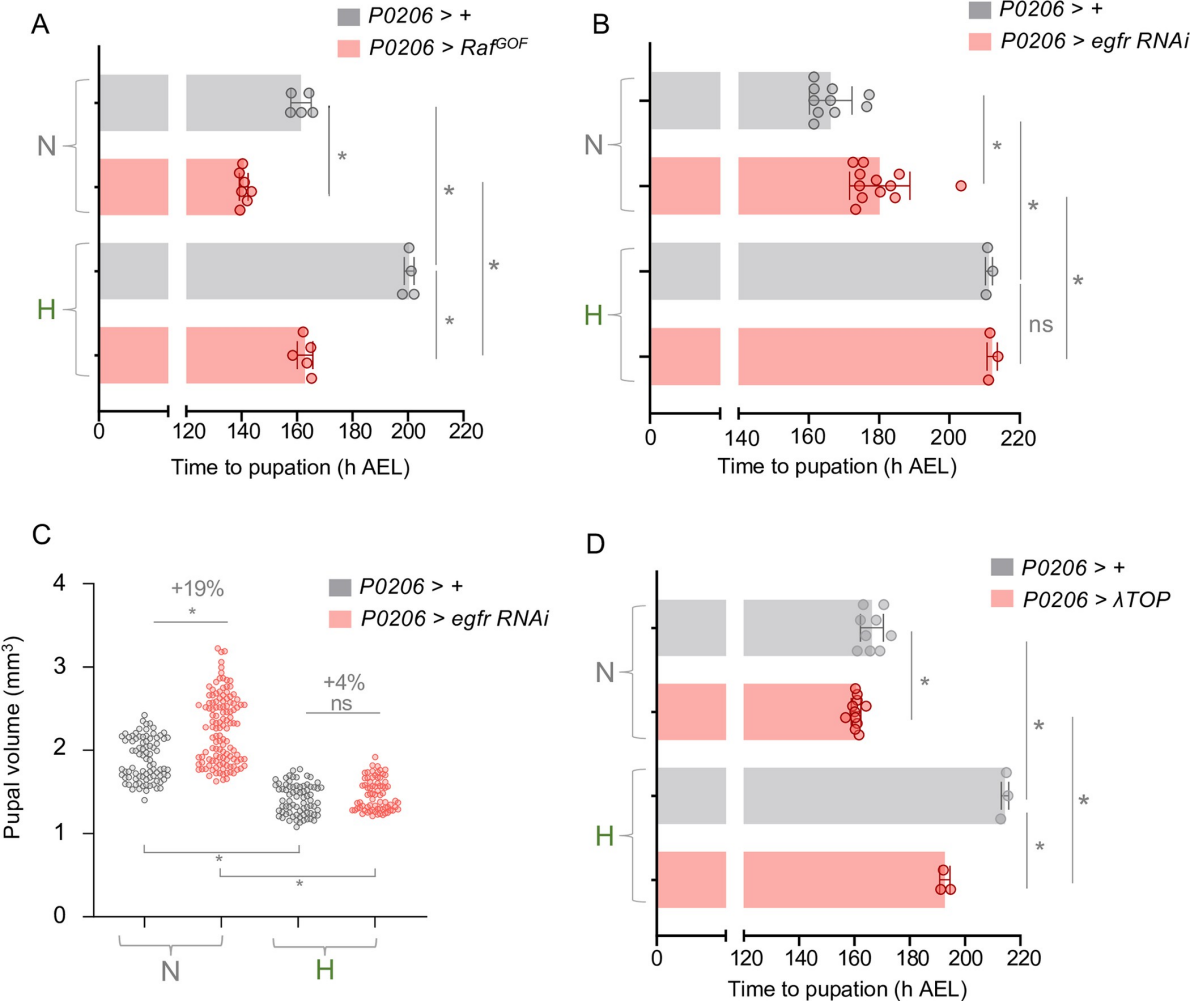
Hypoxia induces a maturation delay by suppressing EGFR/MAPK signaling. **(A)** Mean time to pupation of larvae, either *P0206 >* + or *P0206 > Raf*^*GOF*^, reared in either normal oxygen conditions throughout development (‘N’) or shifted to 5% O_2_ at 120 h AEL (‘H’). n (# of vials of 30 larvae) ≥ 3 per condition. Bars represent mean +/- SEM with individual data points plotted as symbols. **(B)** Mean time to pupation of larvae, either *P0206 >* + or *P0206 > egfr-RNAi*, reared in either normal oxygen conditions throughout development or shifted to 5% O_2_ at 120 h AEL. n (# of vials of 30 larvae) ≥ 3 per condition. Bars represent mean +/- SEM with individual data points plotted as symbols. **(C)** Pupal size of animals, either *P0206 >* + or *P0206 > egfr-RNAi*, reared in normoxia or hypoxia from 120 h AEL. Each data point represents body size measured for one animal. n (# of pupae) = 86 (N *P0206 >* +), 123 (N *P0206 > egfr-RNAi*), 75 (H *P0206 >* +), 74 (H *P0206 > egfr-RNAi*). **(D)** Mean time to pupation of larvae, either *P0206 >* + or *P0206 > λTOP*, reared in either normal oxygen conditions throughout development or shifted to 5% O_2_ at 120 h AEL. n (# of vials of 30 larvae) ≥ 3 per condition. Bars represent mean +/- SEM with individual data points plotted as symbols. * denotes p < 0.05. Also see S7 Fig.

Stimulation of the Ras/ERK signaling pathway in the PG is the key trigger for inducing the late larval pulse of ecdysone synthesis required for pupariation [8,61,62]. We therefore examined the effects of manipulating Ras/ERK signaling in the PG on the hypoxia-induced delay in larval maturation. We found that PG expression (using *P0206-Gal4*) of an activated form of Raf kinase (*UAS-Raf*^*GOF*^) led to pronounced acceleration in the time to pupariation in normoxic larvae (Fig 5A). This result is consistent with previous work showing that genetic activation of Ras/ERK signaling in the PG can induce precocious pupariation by accelerating the onset of the late larval ecdysone pulse [61,62]. Interestingly, we found that PG expression of *UAS-Raf*^*GOF*^ using *P0206-Gal4* also accelerated the time to pupariation in hypoxic larvae (Fig 5A). This accelerated development was even more pronounced when we expressed *Raf*^*GOF*^ with a different PG driver (*spok-Gal4)* and, in this case, the hypoxia-mediated delay in development was almost completely abolished with hypoxia exposed animals pupating only slightly later than normoxic animals (S7A Fig).

A recent paper showed that signalling through Epidermal Growth Factor RTK (EGFR) is the main contributor to Ras/ERK stimulation in the PG [15]. Hence, we explored whether modulation of EGFR affects the hypoxia-mediated delay in pupariation. We found that RNAi-mediated knockdown of EGFR using *phm-Gal4* prevented pupariation, as previously reported (S7B Fig), making it difficult to assess effects of hypoxia on pupariation in these larvae. However, we saw that EGFR knockdown with an alternate weaker PG driver, *P0206-Gal4*, led to a delay in development in normoxic animals and an increase in final pupal size, consistent with the role for EGFR signaling in the timing of maturation (Fig 5B and 5C). Interestingly, the delay to pupariation in hypoxia-exposed animals was not further exacerbated by EGFR knockdown in the PG (Fig 5B) and we saw that the EGFR knockdown animals raised in hypoxia showed no significant difference in final pupal size compared to controls (Fig 5C). This lack of additive effects of EGFR knockdown and hypoxia on delayed development suggest that hypoxia may delay larval maturation by suppressing EGFR signaling. To test this further we examined the effects of overexpression of a constitutively active form of the EGFR in the PG and found that this led to a partial reversal of the hypoxia-mediated delay in development (Fig 5D). Together, these results suggest that one way that hypoxia delays larval maturation is by decreasing EGFR-mediated signalling in the PG. Signalling through two other RTKs, Alk and Pvr, in the PG has also been shown to contribute to Ras/ERK activation and the control of ecdysone synthesis and developmental timing [63,64]. However, we found that RNAi-mediated knockdown of either of these RTKs had little effect on developmental timing (S7C and S7D Fig). We also saw that hypoxia had no effect on the mRNA levels of *pvf2* or *pvf3* (S7E Fig), two ligands that have been shown to be expressed from different cells and tissues including hemocytes, neurons and the PG [63,64], to control ecdysone production and larval maturation by stimulating the Pvr receptor on PG cells. These results suggest that suppression of either Alk or Pvr signaling is unlikely to contribute to the hypoxia-induced delay in larval maturation.

**Fig 6:**
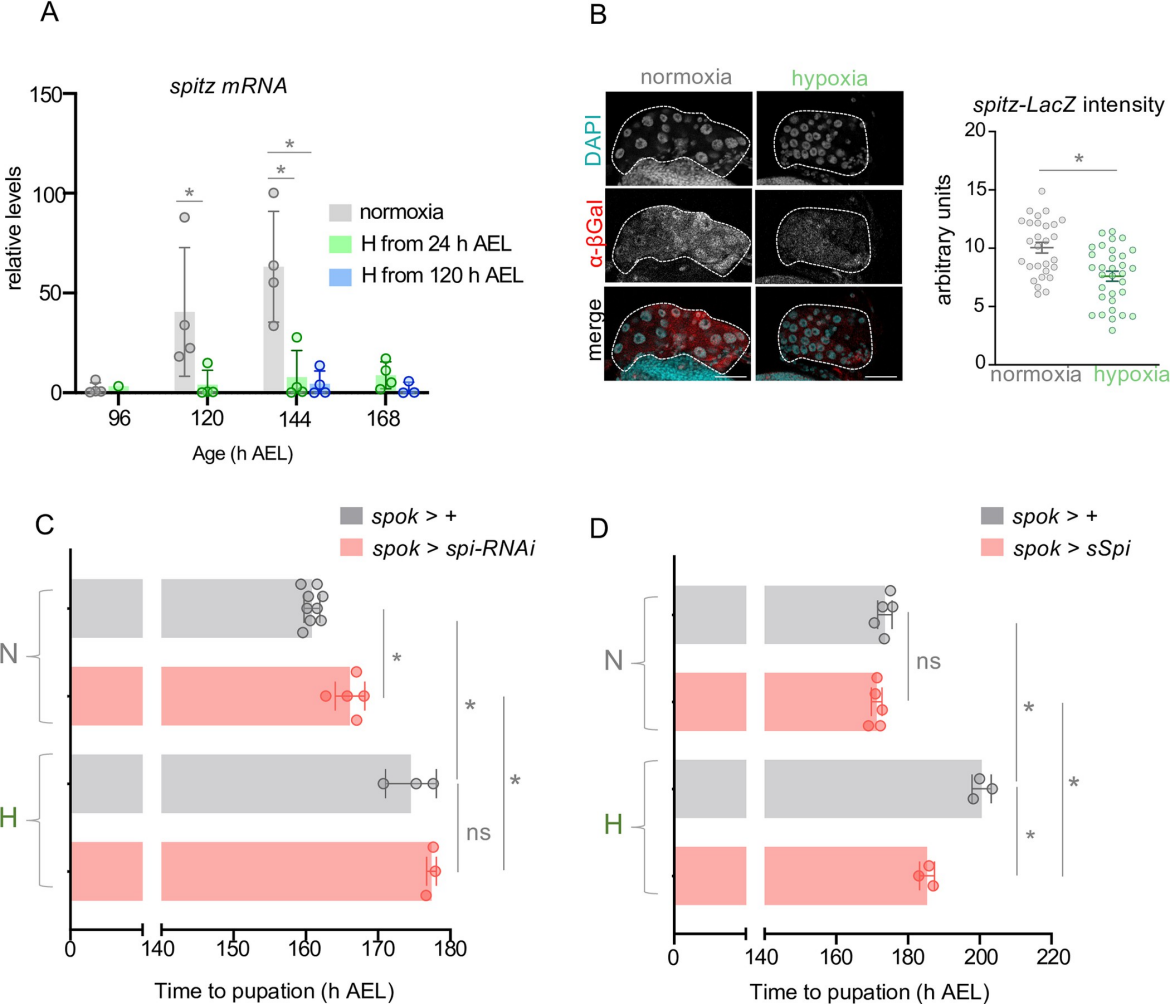
Hypoxia supresses spitz expression to delay EGFR/MAPK-dependent maturation. **(A)** Relative mRNA levels (normalized to Rpl32) of EGF ligand *spitz* from whole-larvae qRT-PCR of *w*^*1118*^ larvae reared in ambient oxygen or in 5% O_2_ from either 24 h AEL or 120 h AEL. n (# of independent samples) ≥ 3 per condition, except for hypoxic larvae at 96 h AEL. **(B)** Representative confocal micrographs and immunofluorescence quantification for anti-beta-galactosidase immunostaining of PGs (indicated with dashed outline) from 140 h AEL *spitz-LacZ* larvae, either reared in normoxia throughout development or in hypoxia from 120 h AEL. Scale bar indicates 50 μm. **(C)** Mean time to pupation of larvae, either *spok >* + or *spok > spitz-RNAi*, reared in either normal oxygen conditions throughout development (‘N’) or shifted to 5% O_2_ at 120 h AEL (‘H’). n (# of vials of 30 larvae) ≥ 3 per condition. Bars represent mean +/- SEM with individual data points plotted as symbols. **(D)** Mean time to pupation of larvae, either *spok >* + or *spok > sSpi* (expressing cleaved/secreted spitz), reared in either normal oxygen conditions throughout development (‘N’) or shifted to 5% O_2_ at 120 h AEL (‘H’). n (# of vials of 30 larvae) ≥ 3 per condition. Bars represent mean +/- SEM with individual data points plotted as symbols. * denotes p < 0.05. Also see S7 and S8 Figs.

There are two main ligands, vein and spitz, that stimulate EGFR in the PG to promote maturation [15]. Interestingly, we saw an upregulation of *spitz* mRNA in late third instar normoxic larvae, as they approach pupariation, and this increased expression was suppressed in larvae that were either raised in hypoxia from hatching or switched to hypoxia post-CW (at 120 hrs) (Fig 6A). In contrast, we found that hypoxia exposure had no effect on *vein* mRNA levels (S7F Fig). In normal development, PG-derived Spitz functions in an autocrine manner to stimulate the EGFR-dependent control of larval maturation. We therefore specifically examined whether hypoxia suppresses Spitz levels in the PG. To do this, we made use of a *spitz-LacZ* enhancer trap line that expresses beta-galactosidase under the control of the *spitz* gene promoter. Using this line, we found that PG LacZ levels were reduced in larvae switched to hypoxia post-CW (at 120 hrs) compared to normoxic age-matched controls (Fig 6B). These results suggest that one way that hypoxia might suppress ecdysone production in the PG is by preventing the late larval stage upregulation of spitz-induced EGFR signaling. The hypoxia-induced decrease in PG spitz-LacZ levels (Fig 6B) was not as strong as the suppression of whole-body spitz mRNA (Fig 6A). This suggests that hypoxia may be regulating Spitz in other tissues. We therefore examined the effects of manipulating PG Spitz levels in different tissues on the hypoxia-induced delay in maturation. We hypothesized that if hypoxia delays larval maturation by suppressing the expression of spitz in a specific tissue(s) then RNAi-mediated knockdown of spitz in this tissue(s) should induce a delay in maturation in normoxic animals that mimics the effects of hypoxia and is not further enhanced by hypoxia exposure. We began by examining the PG since we observed a decrease in spitz-LacZ expression in this tissue. We found that RNAi-mediated knockdown of Spitz in the PG mimicked the effects of hypoxia and delayed pupariation in normoxic animals, as described before (Fig 6C). Moreover, we saw that PG-specific spitz knockdown did not add to the hypoxia-mediated delay in larval maturation, suggesting that this delay may rely on reduced Spitz signalling (Fig 6C). To test this further, we examined the effects of overexpression of Spitz in the PG. We found that PG-specific overexpression of Spitz had little effect on developmental timing in normoxia (Fig 6D). In contrast, Spitz overexpression led to a strong reversal of the hypoxia-induced developmental delay to a similar extent as that seen with 20E feeding (Fig 5D). Spitz is also expressed in several other secretory tissues such as neurons, imaginal discs, the intestine, and the fat body (flyatlas2.org). We therefore examined the effects of suppressing spitz expression in these tissues. We found that RNAi-mediated knockdown of Spitz in imaginal tissues (*esg-Gal4)* neurons (using *elav-Gal4*), or the intestine (*mex-Gal4*) had no effect on pupariation in normoxia and did not affect the hypoxia-mediated delay in larval maturation (S8A–S8C Fig). Interestingly, fat body knockdown of spitz led to a pronounced (~80hrs) delay in pupariation in normoxic larvae that was not further exacerbated by hypoxia exposure (S8D Fig). However, when we measured *Spitz* mRNA levels in isolated fat bodies, we saw no change in hypoxia-exposed larvae (S8E Fig), suggesting that hypoxia is likely not delaying larval maturation by suppressing the expression of fat-body derived Spitz. Together our results suggest hypoxia delays pupariation by suppressing Spitz expression in the PG and perhaps other tissues to blunt the EGFR-induced production of the maturation-inducing pulse of ecdysone.

## Discussion

**Fig 7:**
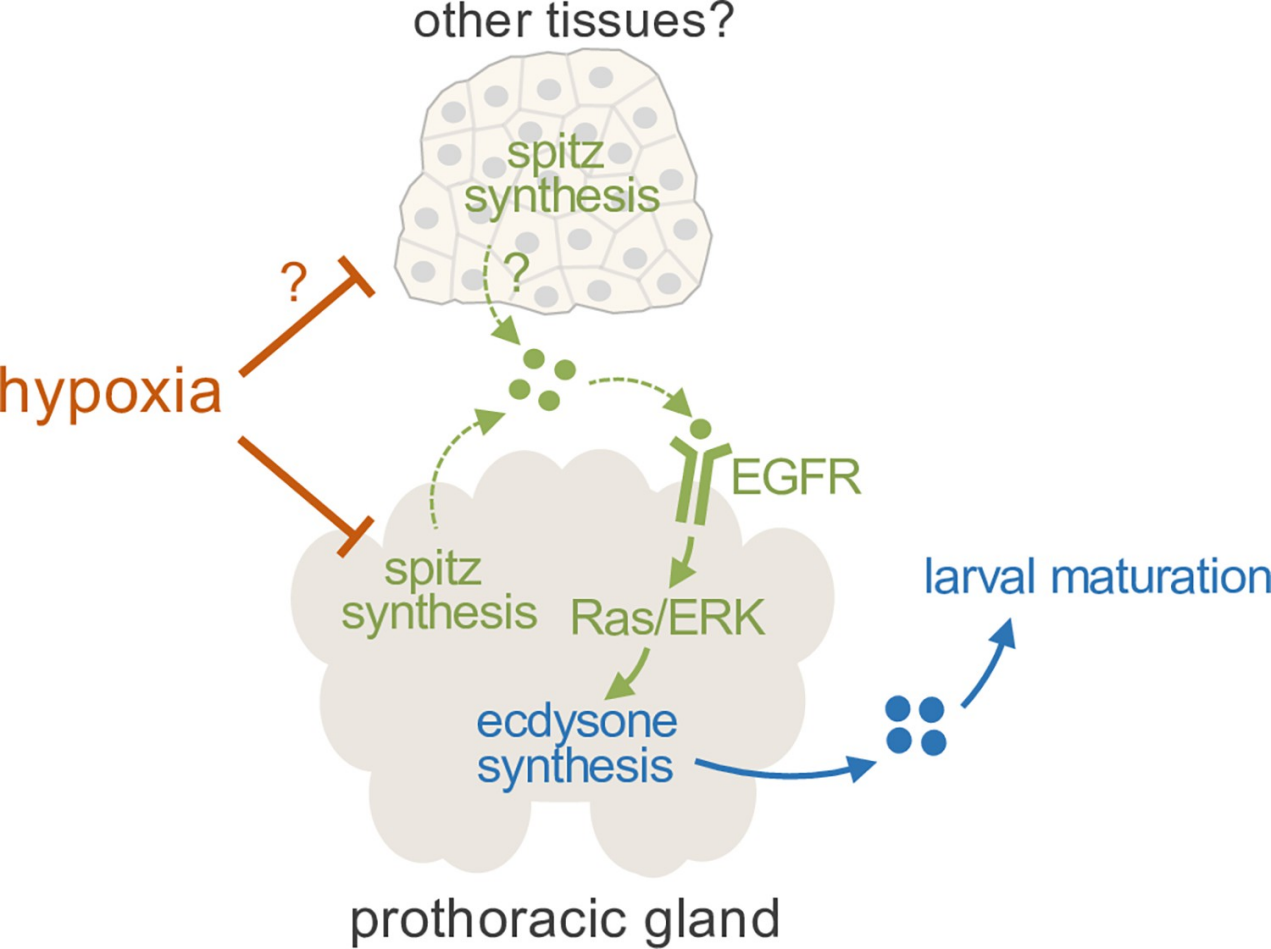
Model for hypoxia-dependent developmental delay. Suppression of ecdysone production by hypoxia at the end of the larval period is at least partially through suppressed EGF ligand (spitz) production by the prothoracic gland (PG). It is also possible that hypoxia additionally suppresses spitz production by distal tissues which might signal to the PG. The result is a delay in the metamorphosis-triggering pulse of ecdysone and subsequent delay in larval maturation.

In this paper, we describe a mechanism by which *Drosophila* larvae delay their maturation in hypoxia. We show that hypoxia can slow early larval growth and development to CW but can also act post-CW to suppress ecdysone biosynthesis in the PG. This post-CW effect involves dampening the production of the EGF ligand, spitz, which normally is needed to activate EGFR signalling, a major stimulator of ecdysone biosynthesis in the PG (Fig 7).

The delay in attaining CW in hypoxic larvae is similar to that seen when larvae are raised on low-nutrient food. The insulin/TOR signalling pathways are the main pathways that couple dietary nutrients to control tissue and body growth. When nutrients are limited, insulin/TOR signaling is reduced and larval growth is slowed [18,32]. The delayed attainment of CW caused by hypoxia exposure in early larval development is most likely due to a similar lowering of systemic insulin/TOR. For example, hypoxia has been shown to promote HIF-1-alpha-independent inhibition of TOR signaling [49,50], suppress systemic insulin signaling by reducing insulin secretion from the brain [48,49], and upregulate expression of the insulin antagonist, ImpL2 [47]. These suppressive effects of hypoxia on insulin/TOR signaling would also persist post CW to reduce late larval growth and hence lead to smaller body size compared to normoxic larvae.

However, an important finding in our work is that hypoxia can also act at the post-CW stage late in larval development to delay maturation by suppressing ecdysone synthesis. Our results are similar to a previous report that examined the effects of moderate hypoxia (10% oxygen) and showed that hypoxia exposure delayed the maturation-inducing ecdysone pulse at the end of the larval period [46]. Interestingly this study also found that hypoxia exposure early in the third instar led to elevated hemolymph ecdysone titers and a subsequent study reported that this elevated ecdysone signaling led to reduced body growth by inducing expression of ImpL2, a negative regulator of systemic insulin signaling [47]. These results suggest that hypoxia exposure may have different effects on ecdysone depending on the stage of larval development. It is also possible that the effects of hypoxia are determined by the level of oxygen—moderate hypoxia may elevate baseline ecdysone titers whereas stronger hypoxia suppresses ecdysone synthesis and signaling. Indeed, we saw that 5% oxygen suppressed the expression of both ecdysone synthesis genes and ecdysone target genes prior to the maturation-inducing peak of ecdysone synthesis at the end of the larval period. The notion of hypoxia having differential effects on growth signaling pathways depending on the level of oxygen is also supported by our previous work where we found that TOR kinase activity is suppressed between 1% and 6% oxygen but is unaffected at either higher or lower oxygen levels [50]. Further studies will be required to define if and how varying oxygen levels influence ecdysone signaling.

We also found that the suppression of ecdysone synthesis genes was equally strong when larvae were either raised in hypoxia from hatching or transferred to hypoxia post-CW, suggesting a specific effect of hypoxia on the late larval events that lead to ecdysone biosynthesis. In this post-CW context, there is a difference between the effects of hypoxia and nutrient restriction on maturation. When starved post-CW, larvae can successfully trigger the late ecdysone pulse and mature to the pupal stage. This is thought to be because they have reached a sufficient mass and level of stored nutrients to support metamorphosis during the non-feeding pupal stage [8,9]. Indeed, critical weight is operationally defined as the point at which starvation does not lead to growth arrest and failure to maturation. The nutrient regulation of this CW checkpoint has been shown to rely on stimulation of insulin/TOR signaling in the PG [34–36], and genetic stimulation of these pathways in the PG is sufficient to reverse the delay in development caused by nutrient limitation. However, we found that activation of TOR or knockdown of FOXO in the PG did not accelerate development in hypoxia and hypoxia exposure after CW produced a substantial delay in development. Our findings suggest that larvae use different maturation strategies to cope with nutrient deprivation and low oxygen, which could reflect distinct metabolic strategies in response to fluctuations in these two environmental cues. For example, while starvation leads to lipid depletion [65], we previously showed that larvae increase their lipid levels in hypoxia [50]. The hypoxia-induced accumulation in fat body lipids is needed to support subsequent pupal development [50]. Therefore, it is possible that when exposed to hypoxia, larvae need to extend their growth and feeding period to ensure that they accumulate these important nutrient stores. Failure to delay development in hypoxia may therefore impact proper metamorphosis. In addition, since we have also previously shown that hypoxia exposure during the larval period can exert subsequent effects on adult metabolism, stress tolerance and lifespan [66], any defects in properly delaying development in low oxygen could impact subsequent adult fitness.

Our data point to decreased Spitz expression and suppression of EGFR/Ras/ERK in the PG as one way that hypoxia delays larval maturation. Ptth/Torso signaling is well established as a trigger for stimulating ERK-dependent ecdysone synthesis in the PG [12,13,62,63]. However, our data suggest that hypoxia and Ptth/Torso suppression function independently to delay development—we saw that larvae with reduced Ptth/Torso signaling still extend their developmental period in hypoxia, and this extension is greater than that seen with Ptth/Torso inhibition in normoxia. We also found little effect of knockdown of either Alk or Pvr on developmental timing, suggesting that they are unlikely to be involved in the hypoxia-induced delay in development. These results contrast with recent studies that have implicated both RTKs in the control of larval maturation [63,64]. These differences may reflect subtle differences in nutrient conditions between our work and these previous studies, especially since the effects of Pvr signalling on developmental timing are regulated by nutrients [64]. Our results also suggest that hypoxia suppresses Spitz expression in tissues other than the PG, since we saw a strong reduction in whole-body Spitz mRNA levels. However, when we tested some candidate tissues that are known to express Spitz, we found that only knockdown of Spitz in the fat body led to a delay in larval maturation but also that hypoxia didn’t alter fat body *Spitz* mRNA levels. Further studies will be required to explore which other tissues hypoxia may act on to suppress Spitz expression and whether Spitz signals from these tissues to control larval maturation.

How might hypoxia suppress Spitz expression? Our data suggest that the effects are independent of HIF-1 alpha/sima, the best-described mediator of hypoxia-induced changes in gene expression. Although previous studies have shown that HIF-1 alpha plays important roles in other *Drosophila* larval tissues to control adaptations to hypoxia [49,57–60,67], our results add to the body of work indicating that HIF-1 alpha-independent mechanisms are also important in controlling organismal adaptations to hypoxia [50,55,59]. The upregulation of *spitz* mRNA expression in the PG at the end of the larval period is triggered by an initial pulse of ecdysone signaling through the ecdysone receptor (EcR) [15]. These nuclear hormone receptors bind DNA and directly control gene expression. Hence, it is possible that hypoxia can antagonize EcR-dependent expression of Spitz. Interestingly, in *C elegans*, hypoxia has been shown to modulate nuclear hormone receptor activity to antagonize EGF/ERK signaling [68]. Hypoxia could also control PG gene expression via alpha-ketoglutarate-dependent dioxygenases, which include several chromatin modifiers [69]. These use oxygen in their reaction cycle and are directly regulated by hypoxia [70, 71]. Hypoxia can also stimulate production of L-2-hydroxyglutarate [72], which can inhibit the alpha-ketoglutarate-dependent dioxygenases. One member of this family of chromatin modifiers, KDM5, functions in the PG to control developmental timing in *Drosophila* [73], while mammalian members of this family have been shown to control the expression of different cytokines and growth factors [74, 75].

Our study has implications for understanding how low oxygen levels can affect development in normal and disease conditions. Hypoxia suppression of EGF signaling is conserved in other organisms [68, 76], and altered expression of EGFR signaling components has been reported both in human populations living in high altitudes [77] and in *Drosophila* populations which have undergone multiple generations of selection for hypoxia tolerance [78]. Also, hypoxia can modulate steroidogenesis and steroid functions in many animals [79–82], and diseases that can result in reduced systemic oxygen levels in humans—such as chronic obstructive pulmonary disease, sleep apnea or asthma—can impact steroid-induced development and sexual maturation [83–85]. Thus, the mechanisms that we describe here in *Drosophila* may reflect more general mechanisms by which low oxygen can affect animal development.

## Materials and methods

### *Drosophila* food and genetics

The experimental larvae were raised on food consisting of 150 g agar, 1600 g cornmeal, 770 g Torula yeast, 675 g sucrose, 2340 g D-glucose, and 240 mL of an acid mixture (propionic acid/phosphoric acid) for every 34 L of water, and maintained at 25°C. For starvation experiments, larvae were switched from standard food to vials containing only agar/water. For all GAL4/UAS experiments, experimental larvae were those obtained from crossing GAL4 lines with the appropriate UAS line. Control animals were obtained by mating the relevant GAL4 line with flies of the same genetic background as the relevant experimental UAS transgene line.

### Drosophila stocks

For a complete list of fly stocks used, refer to S1 Table.

### Hypoxia exposure

Vials containing larvae were placed in an airtight chamber into which a gas mixture of 5% oxygen and 95% nitrogen was continuously flowed. The rate of flow was controlled using an Aalborg model P gas flow meter. The developmental age of exposure is indicated in individual figures–either 24, 72, 96 or 120 h AEL. For developmental timing experiments, larvae were left in hypoxia to pupate until no further pupation was observed for 24 hours after the initial onset of pupation was observed in all vials. During this time, developmental timing was measured by counting pupae through the transparent walls of the hypoxia chamber.

### Measurement of *Drosophila* developmental timing

For each experimental cross, females were allowed to lay eggs on a grape juice/agar plate smeared with yeast paste for four hours. 24hours later, newly hatched larvae were transferred to vials containing our standard lab fly food. 30 larvae were transferred per vial and the number of larvae that pupated in each vial were subsequently counted twice a day. A minimum of three vials of 30 larvae were used to calculate the average time to pupation and percent pupation for each experimental group.

### Pupal volume measurements

Pupae were positioned ventral side-down on a petri dish. Images of pupae were captured with the ZEISS SteREO Discovery.V8 microscope and ZEN imaging software (blue edition) at 1.25x magnification. From these images, the ZEN software was used to measure the width and length of each pupa (in μm). These values were then entered into a mathematical formula for the volume of an ellipsoid (4/3π x L/2 x W). Each value resulting from this calculation constituted the pupal volume of one animal.

### Quantitative real-time PCR (qRT-PCR)

For whole larvae experiments, total RNA was extracted from larvae in groups of 8 using TRIzol according to manufacturer instructions (Invitrogen; 15596–018). For ring gland experiments, samples of 10–12 ring glands were collected. For fat body experiments, 8 animal equivalents of fat body were collected per sample. RNA samples were then DNase-treated with Ambion Turbo DNase according to manufacturer’s instructions (Ambion; 2238 G) and reverse transcribed with Superscript II (Invitrogen; 100004925). Resultant cDNA was used as a template for qRT–PCR (ABI 7500 real time PCR system using SyBr Green PCR mix) primer pairs for genes of interest. PCR data were normalized to levels of *rpl32* or *5SrRNA* both of which are unaffected by hypoxia. For complete list of primers and sequences, refer to S2 Table.

### Starvation treatment

*w*^*1118*^ larvae were collected at 24 h AEL and transferred into regular food vials (30 larvae per vial). At 72, 96 and 120 h AEL, larvae were removed from these vials by suspending them in 20% sucrose and transferred to vials containing either regular food or an equal volume of 0.8% agar.

### Determination of age at critical weight attainment in hypoxia and normoxia

*w*^*1118*^ larvae were collected and transferred into regular food vials (30 larvae per vial). These vials were either placed into hypoxia to develop or left at ambient oxygen. At 96, 100, 112, 116, 120, 124 and 136 h AEL, larvae were transferred to 0.8% agar as in the above protocol and left to develop in ambient oxygen. The resulting percent pupation was calculated and plotted as a function of the age at starvation for each vial. A minimum of 3 vials of 30 larvae were used to calculate the mean percent pupation for each condition at each starvation time point. The point on the x-axis (age of starvation in h AEL) on the graph where >50% mean pupation was achieved was defined as the age at critical weight attainment for each treatment group (normoxia or hypoxia).

### 20-hydroxyecdysone feeding

*w*^*1118*^ larvae were collected at 24 h AEL and transferred into vials containing food supplemented either with 20-hydroxyecdysone (Sigma-Aldrich CAS number 5289-74-7) dissolved in 95% ethanol (final 20E concentration of 0.3 mg/mL) or an equal volume of 95% ethanol alone.

### Immunostaining and microscopy

Larvae were inverted and fixed in 8% formaldehyde (Electron Microscopy Science, Hatfield, U.S.A.) in PBS at room temperature for 30 minutes, then blocked for 2 hours in 1% BSA in PBS/0.1% Triton-X 100 at room temperature. Blocked samples were then incubated at 4°C overnight in primary antibody (rabbit anti-beta-galactosidase, Jackson ImmunoResearch Laboratories) dissolved at a 1:200 dilution in 1% BSA in PBS/0.1% Triton-X 100. Tissues were then incubated in secondary antibody (Alexa Fluor 568 (Molecular Probes) goat anti-rabbit secondary antibody (1:2000). DNA was visualized using Hoechst 33258 (Invitrogen, 1:10 000). Tissues were then dissected out and mounted on glass slides using Vectashield mounting media. Confocal micrographs were captured using a ZEISS confocal microscope LSM 880.

### Quantification of Spitz expression in PG cells

Confocal slices of ring glands from *spitz-LacZ* larvae stained for β-galactosidase were used for analysis in Fiji ImageJ2 (Version 2.3.0). β-galactosidase staining intensity was measured by measuring the LacZ fluorescence intensity within a fixed area at four random regions within each PG analyzed.

### Ecdysteroid quantification

To extract and quantify ecdysteroids, a modified protocol of that in [64] was employed. Samples of 10–12 larvae were collected in Eppendorf tubes and snap frozen on dry ice. They were then homogenized in 0.3 mL of 100% methanol using a handheld motorized pestle and centrifuged at 15000 rpm at room temperature. The supernatant was then transferred to a new Eppendorf tube and this process of homogenization in 0.3mL of methanol followed by centrifugation and supernatant transfer was repeated three times, yielding 0.9mL per sample. For each sample, half of this volume was transferred to a new tube and evaporated on a SpeedVac machine for roughly 90 minutes or until the dried lipid fraction was visible on the bottom of the tube. A competitive enzyme-linked immunosorbent assay (ELISA) was then performed using these samples according to manufacturer instructions (Bertin Pharma, no. A05120; 96 wells) and the absorbance at 410nm was measured with a microplate reader (SpectraMax M2e from Molecular Devices).

### Statistical analyses

The mean times to pupation and pupal volume measurements were analyzed by two-way ANOVA followed by unpaired Student’s t-test with Welch’s correction for unequal variance, using an alpha value of 0.05. For qRT-PCR, unpaired Student’s t-test with Welch’s correction for unequal variance was employed for pairwise comparisons. Graphs and statistical analyses were produced using GraphPad Prism 9.0.0 for Windows by GraphPad Software (San Diego, California USA). Two ANOVA results are presented in S3 Table.

## Supporting information

S1 Fig (related to Fig 1). **(A)** Pupal volume of *w*^*1118*^ larvae reared in normoxia throughout development (‘N’) or in hypoxia from 24 h AEL (‘H’). n (# of pupae) = 112 (normoxia) and 83 (hypoxia) **(B)** % survival to the pupal stage of *w*^*1118*^ larvae reared in normoxia throughout development or in hypoxia from 24 h AEL. Each data point represents the average calculated from a vial of 30 larvae. n (# of vials of 30 larvae) ≥ 3 per condition. Bars represent mean +/SEM with individual data points plotted as symbols. * denotes p < 0.05; ns denotes non–significant.
(PDF)

S2 Fig (related to Fig 2). **(A)** % survival to the pupal stage of *w*^*1118*^ *larvae* either starved or exposed to 5% O_2_ at the indicated larval age. Each data point represents the average calculated from a vial of 30 larvae. n (# of vials of 30) ≥ 3 per condition. Bars represent mean +/SEM with individual data points plotted as symbols. **(B)** Pupal volume of *w*^*1118*^ *larvae* either starved or exposed to 5% O_2_ (‘H’) at the indicated larval age. n (# of pupae) = 148 (normoxia), 107 (H @ 72 h AEL), 91 (H @ 96 h AEL), 107 (H @ 120 h AEL), 62 (starvation @ 120 h AEL). **(C)** Relative mRNA levels (normalized to Rpl32) of ecdysone biosynthetic genes *phantom* (*phm*) and *spookier* (*spok*) from ring gland-specific qRT-PCR of *w1118* larvae reared in ambient oxygen or in 5% oxygen from 120 h AEL (*i*.*e*. post-CW). n (# of independent samples) ≥ 4. Bars represent mean +/SEM with individual data points plotted as symbols. * denotes p < 0.05; ns denotes non–significant. **(D)** Pupal volume of *w*^*1118*^ *larvae* either raised at ambient oxygen or 5% oxygen from 120 h AEL (‘H’). n (#of pupae) = 68 (N, EtOH), 69 (N, 20E), 137 (H, EtOH), 127 (H, 20E). * denotes p < 0.05; ns denotes non–significant.
(PDF)

S3 Fig (related to Fig 2). **(A)** Average time to pupation of larvae, either *P0206 >* + or *P0206 > Rheb*, reared in either normal oxygen conditions throughout development or shifted to 5% O_2_ at 120 h AEL. n (# of vials of 30 larvae) ≥ 3 per condition. * denotes p < 0.05, N.S. denotes not significant. **(B)** Pupal size of animals reared in normoxia or hypoxia from 120 h AEL. Each data point represents body size measured for one animal. n (# of pupae) = 95 (N, *P0206>+*), 66 (N, *P0206>Rheb*), 60 (H, *P0206>+*), 59 (H, *P0206>Rheb*). * denotes p < 0.05. **(C)** Average time to pupation of larvae, either *spok >* + or *spok > foxo-RNAi*, reared in either normal oxygen conditions throughout development or shifted to 5% O_2_ at 120 h AEL. n (# of vials of 30 larvae) ≥ 3 per condition. * denotes p < 0.05, N.S. denotes not significant. Bars represent mean +/SEM with individual data points plotted as symbols. **(D)** Confocal micrographs of ring glands from larvae, either *P0206>UAS-GFP* or *spok>UAS-GFP*, reared in normoxia or 5% oxygen from 120 hAEL and dissected at 144 hAEL.
(PDF)

S4 Fig (related to Fig 3). **(A)** Relative mRNA levels of HIF-1α target gene, *fatiga* from whole-larvae qRT-PCR of larvae, either *da>+* or *da>sima-i*, reared in ambient oxygen or in 5% O_2_ from 24 h. n (# of independent samples) ≥ 3 per condition. **(B)** Pupal volume of *w*^*1118*^ *larvae* either raised at ambient oxygen (‘N’) or 5% oxygen from 120 h AEL (‘H’). n (# of pupae) = 102 (N, *phm > UAS-Dicer*),100 (N, *phm>UAS-Dicer*, *sima-i*),60 (H, *phm > UAS-Dicer*), 66 (H, *phm>UAS-Dicer*, *sima-i*) **(C)** Mean time to pupation of larvae, either *elav>+* or *elav>sima-i*, reared in either normal oxygen conditions throughout development (‘N’) or shifted to 5% O_2_ at 120 h AEL (‘H’). n (# of vials of 30 larvae) ≥ 3 per condition. * denotes p < 0.05, N.S. denotes not significant. Bars represent mean +/SEM with individual data points plotted as symbols.
(PDF)

S5 Fig (related to Fig 4). **(A)** Representative images of dilp8-GFP expression in wing discs from 144h AEL larvae reared in either normal oxygen conditions throughout development (‘N’) or shifted to 5% O_2_ at 120 h AEL. Scale bar indicates 50mm. **(B)** Relative mRNA levels of *dilp8* mRNA, from whole-larvae qRT-PCR of larvae reared in ambient oxygen, 5% O_2_ from 24 h AEL or 5% O_2_ from 120 h AEL. Bars represent mean +/SEM with individual data points plotted as symbols. ns denotes not significant. **(C)** Mean time to pupation of larvae, either *da>+* or *da>dilp8iRNAi (2)*, reared in either normal oxygen conditions throughout development (‘N’) or shifted to 5% O_2_ at 120 h AEL (‘H’). n (# of vials of 30 larvae) ≥ 3 per condition. * denotes p < 0.05, ns denotes not significant.
(PDF)

S6 Fig (related to Fig 4). **(A)** Average time to pupation of larvae, either *w*^*1118*^ or *ptth*^*120F2A*^ (an alternative ptth null mutant) reared in either normal oxygen conditions throughout development (‘N’) or shifted to 5% O_2_ at 120 h AEL (‘H’). n (# of vials of 30 larvae) ≥ 3 per condition. Bars represent mean +/SEM with individual data points plotted as symbols. * denotes p < 0.05; ns denotes non–significant. **(B)** Pupal size of animals, either *w*^*1118*^ or mutant for *ptth*, reared in normoxia or hypoxia from 120 h AEL. Each data point represents body size measured for one animal. n (# of pupae) = 150 (N *w*^*1118*^), 150 (N *ptth*^*120F2A*^), 29 (H *w*^*1118*^), 67 (H *ptth*^*120F2A*^). * denotes p < 0.05.
(PDF)

S7 Fig (related to Figs 5 and 6). **(A)** Average time to pupation of larvae, either *spok >* + or *spok > Raf*^*GOF*^, reared in either normal oxygen conditions throughout development (‘N’) or shifted to 5% O_2_ at 120 h AEL (‘H’). n (# of vials of 30 larvae) ≥ 3 per condition. **(B)** Representative images of animals, either *phm >* + or *phm > Egfr-RNAi*¸ reared in normoxia throughout development or shifted to 5% O_2_ at 120 h AEL and imaged at the indicated larval age. In both treatments, *phm > Egfr-RNAi* arrests development prior to pupation. Scale bar = 1 mm. **(C, D)** Average time to pupation of larvae expressing RNAi against receptor tyrosine kinases *Alk* (C) or *Pvr* (D) under the control of *P0206-GAL4* reared in normal oxygen conditions. n (# of vials of 30 larvae) ≥ 3 per condition. **(E, F)** Relative mRNA levels of Pvr ligands *Pvf2* and *Pvf3* (E) and Egf ligand *vein* (F), from whole-larvae qRT-PCR of larvae reared in ambient oxygen, 5% O_2_ from 24 h AEL or 5% O_2_ from 120 h AEL. n (# of independent samples) ≥ 3 per condition. Bars represent mean +/SEM with individual data points plotted as symbols. * denotes p < 0.05; ns denotes non–significant.
(PDF)

S8 Fig (related to Fig 6). **(A-D)** Average time to pupation of larvae of the indicated genotype, reared in either normal oxygen conditions throughout development (‘N’) or shifted to 5% O_2_ at 120 h AEL (‘H’). n (# of vials of 30 larvae) ≥ 3 per condition. *esg-GAL4*, *elav-GAL4*, *mex-GAL4 and r4-GAL4* were used to drive *UAS-spi-RNAi* expression in the imaginal discs, neurons, enterocytes and fat body, respectively. **(E)** Relative mRNA levels of Egf ligand *spitz*, from fat body qRT-PCR of larvae reared in ambient oxygen, 5% O_2_ from 24 h AEL or 5% O_2_ from 120 h AEL. n (# of independent samples) ≥ 3 per condition. Bars represent mean +/SEM with individual data points plotted as symbols. * denotes p < 0.05; ns denotes non–significant.
(PDF)

S1 Table List of fly stocks used in this study. (PDF)

S2 Table List of primers used in this study. (PDF)

S3 Table Summary of Two-Way ANOVA results/. (PDF)
