# Supplementary material for: Hypoxia delays steroid-induced developmental maturation in Drosophila by suppressing EGF signaling
Source: PLoS Genet. 2024 Apr 26;20(4):e1011232. doi: 10.1371/journal.pgen.1011232 (PMC11098494; doi:10.1371/journal.pgen.1011232)
Supplement: S1 Fig — (A) Pupal volume of w1118 larvae reared in normoxia throughout development (‘N’) or in hypoxia from 24 h AEL (‘H’). n (# of pupae) = 112 (normoxia) and 83 (hypoxia) (B) % survival to the pupal stage of w1118 larvae reared in normoxia throughout development or in hypoxia from 24 h AEL. Each data point represents the average calculated from a vial of 30 larvae. n (# of vials of 30 larvae) ≥ 3 per condition. Bars represent mean +/SEM with individual data points plotted as symbols. * denotes p < 0.05; ns denotes non–significant. (PDF) [file pgen.1011232.s001.pdf]

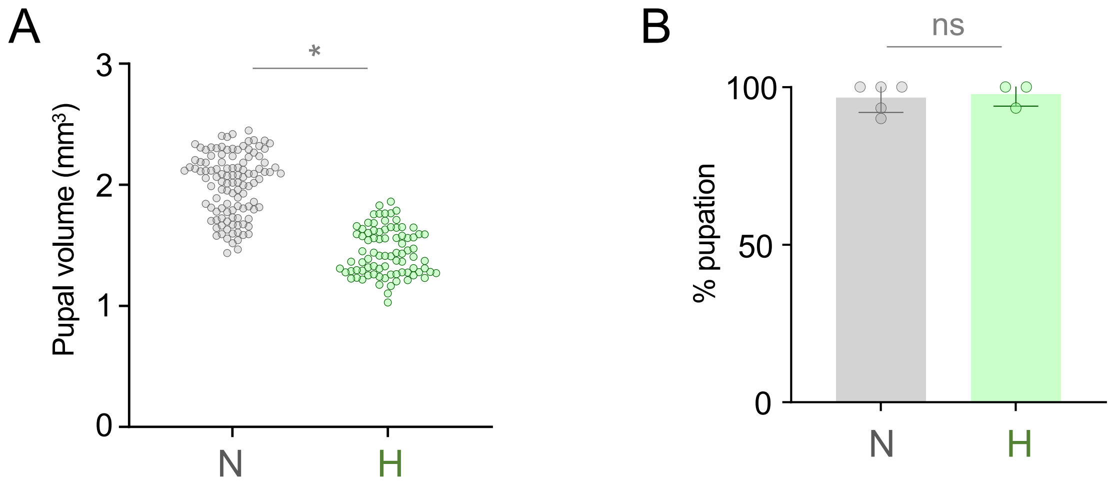

Figure S1 (related to Figure 1). (A) Pupal volume of *w*^{1118} larvae reared in normoxia throughout development (‘N’) or in hypoxia from 24 h AEL (‘H’). n (# of pupae) = 112 (normoxia) and 83 (hypoxia) **(B)** % survival to the pupal stage of *w*^{1118} larvae reared in normoxia throughout development or in hypoxia from 24 h AEL. Each data point represents the average calculated from a vial of 30 larvae. n (# of vials of 30 larvae) ≥ 3 per condition. Bars represent mean +/- SEM with individual data points plotted as symbols. * denotes $p < 0.05$; ns denotes not significant.
