# Supplementary material for: Hypoxia delays steroid-induced developmental maturation in Drosophila by suppressing EGF signaling
Source: PLoS Genet. 2024 Apr 26;20(4):e1011232. doi: 10.1371/journal.pgen.1011232 (PMC11098494; doi:10.1371/journal.pgen.1011232)
Supplement: S2 Fig — (A) % survival to the pupal stage of w1118 larvae either starved or exposed to 5% O2 at the indicated larval age. Each data point represents the average calculated from a vial of 30 larvae. n (# of vials of 30) ≥ 3 per condition. Bars represent mean +/SEM with individual data points plotted as symbols. (B) Pupal volume of w1118 larvae either starved or exposed to 5% O2 (‘H’) at the indicated larval age. n (# of pupae) = 148 (normoxia), 107 (H @ 72 h AEL), 91 (H @ 96 h AEL), 107 (H @ 120 h AEL), 62 (starvation @ 120 h AEL). (C) Relative mRNA levels (normalized to Rpl32) of ecdysone biosynthetic genes phantom (phm) and spookier (spok) from ring gland-specific qRT-PCR of w1118 larvae reared in ambient oxygen or in 5% oxygen from 120 h AEL (i.e. post-CW). n (# of independent samples) ≥ 4. Bars represent mean +/SEM with individual data points plotted as symbols. * denotes p < 0.05; ns denotes non–significant. (D) Pupal volume of w1118 larvae either raised at ambient oxygen or 5% oxygen from 120 h AEL (‘H’). n (#of pupae) = 68 (N, EtOH), 69 (N, 20E), 137 (H, EtOH), 127 (H, 20E). * denotes p < 0.05; ns denotes non–significant. (PDF) [file pgen.1011232.s002.pdf]

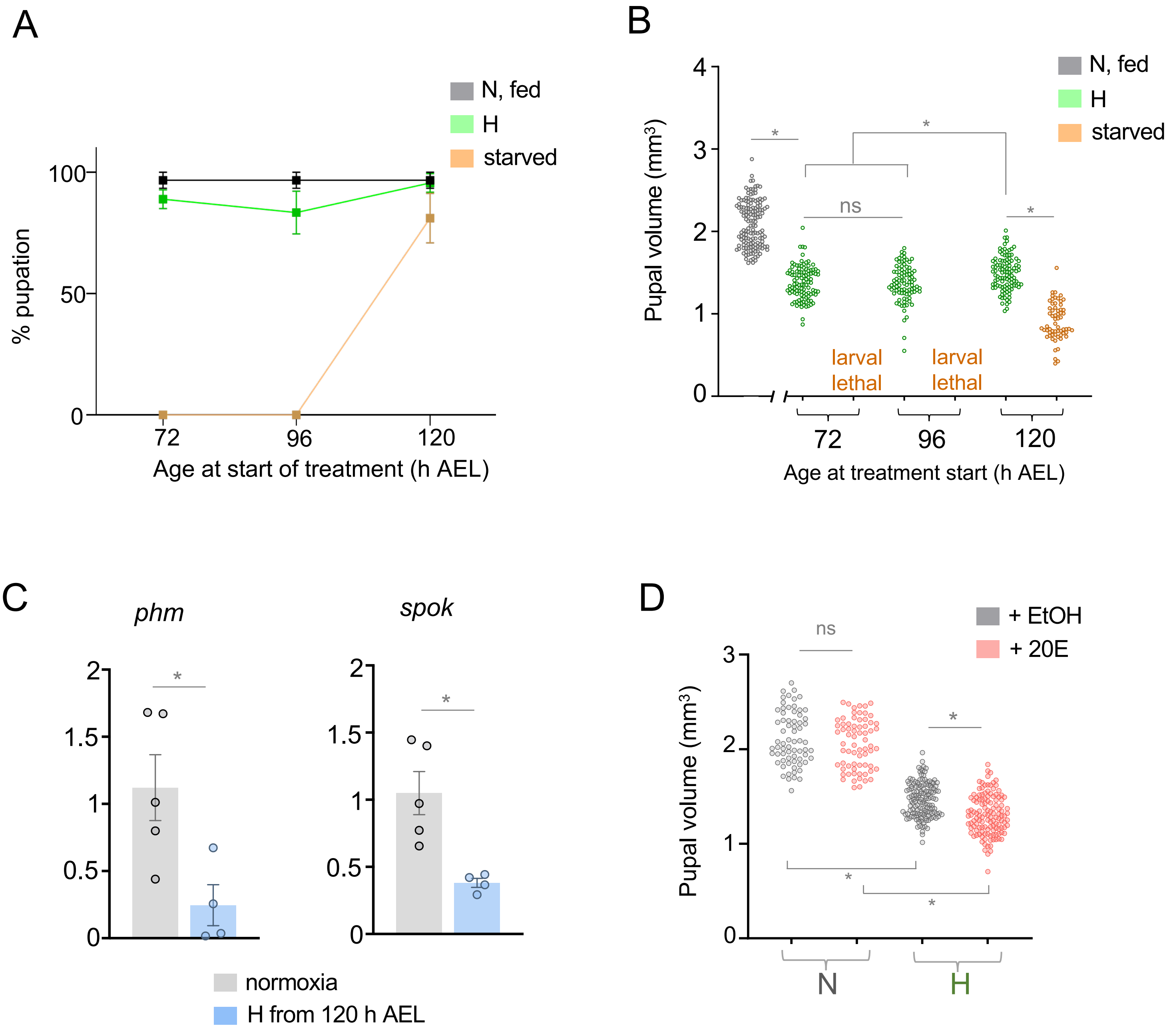

Figure S2 (related to Figure 2). (A) % survival to the pupal stage of *w[1118]* larvae either starved or exposed to 5% O_2 at the indicated larval age. Each data point represents the average calculated from a vial of 30 larvae. n (# of vials of 30) ≥ 3 per condition. Bars represent mean +/- SEM with individual data points plotted as symbols. **(B)** Pupal volume of *w[1118]* larvae either starved or exposed to 5% O_2 ('H') at the indicated larval age. n (# of pupae) = 148 (normoxia), 107 (H @ 72 h AEL), 91 (H @ 96 h AEL), 107 (H @ 120 h AEL), 62 (starvation @ 120 h AEL). **(C)** Relative mRNA levels (normalized to Rpl32) of ecdysone biosynthetic genes *phantom* (*phm*) and *spookier* (*spok*) from whole-larvae qRT-PCR of *w1118* larvae reared in ambient oxygen or in 5% oxygen from 120 h AEL (*i.e.* post-CW). n (# of independent samples) ≥ 4. Bars represent mean +/- SEM with individual data points plotted as symbols. * denotes $p < 0.05$; ns denotes not significant. **(D)** Pupal volume of *w[1118]* larvae either raised at ambient oxygen or 5% oxygen from 120 h AEL ('H'). n (#of pupae) = 68 (N, EtOH), 69 (N, 20E), 137 (H, EtOH), 127 (H, 20E). * denotes $p < 0.05$; ns denotes not significant.
