# Supplementary material for: Hypoxia delays steroid-induced developmental maturation in Drosophila by suppressing EGF signaling
Source: PLoS Genet. 2024 Apr 26;20(4):e1011232. doi: 10.1371/journal.pgen.1011232 (PMC11098494; doi:10.1371/journal.pgen.1011232)
Supplement: S3 Fig — (A) Average time to pupation of larvae, either P0206 > + or P0206 > Rheb, reared in either normal oxygen conditions throughout development or shifted to 5% O2 at 120 h AEL. n (# of vials of 30 larvae) ≥ 3 per condition. * denotes p < 0.05, N.S. denotes not significant. (B) Pupal size of animals reared in normoxia or hypoxia from 120 h AEL. Each data point represents body size measured for one animal. n (# of pupae) = 95 (N, P0206>+), 66 (N, P0206>Rheb), 60 (H, P0206>+), 59 (H, P0206>Rheb). * denotes p < 0.05. (C) Average time to pupation of larvae, either spok > + or spok > foxo-RNAi, reared in either normal oxygen conditions throughout development or shifted to 5% O2 at 120 h AEL. n (# of vials of 30 larvae) ≥ 3 per condition. * denotes p < 0.05, N.S. denotes not significant. Bars represent mean +/SEM with individual data points plotted as symbols. (D) Confocal micrographs of ring glands from larvae, either P0206>UAS-GFP or spok>UAS-GFP, reared in normoxia or 5% oxygen from 120 hAEL and dissected at 144 hAEL. (PDF) [file pgen.1011232.s003.pdf]

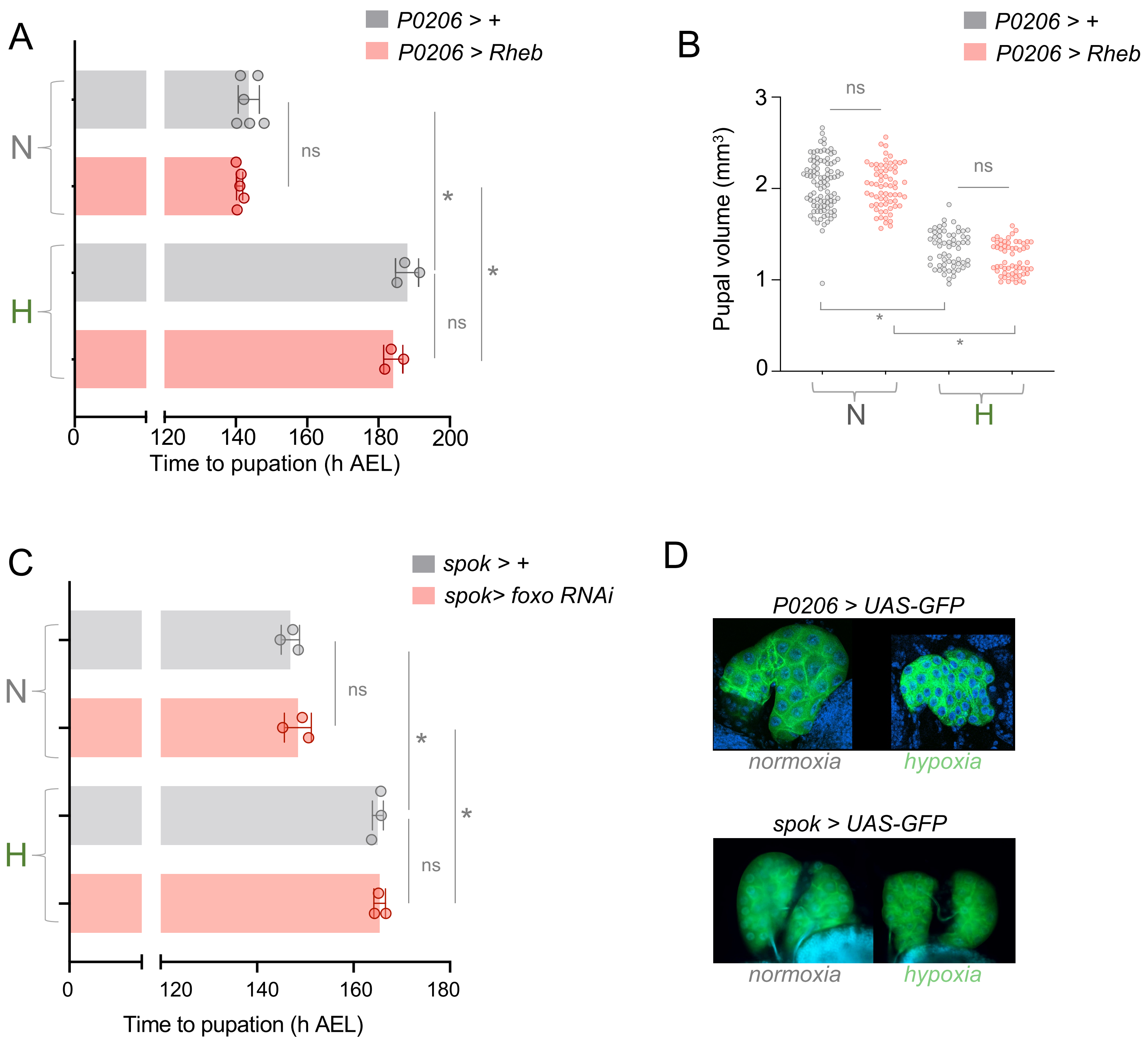

Figure S3 (related to Figure 2). (A) Average time to pupation of larvae, either *P0206 > +* or *P0206 > Rheb*, reared in either normal oxygen conditions throughout development or shifted to 5% O_2 at 120 h AEL. n (# of vials of 30 larvae) ≥ 3 per condition. * denotes $p < 0.05$, N.S. denotes not significant. **(B)** Pupal size of animals reared in normoxia or hypoxia from 120 h AEL. Each data point represents body size measured for one animal. n (# of pupae) = 95 (N, *P0206>+*), 66 (N, *P0206>Rheb*), 60 (H, *P0206>+*), 59 (H, *P0206>Rheb*). * denotes $p < 0.05$. **(C)** Average time to pupation of larvae, either *spok > +* or *spok > foxo-RNAi*, reared in either normal oxygen conditions throughout development or shifted to 5% O_2 at 120 h AEL. n (# of vials of 30 larvae) ≥ 3 per condition. * denotes $p < 0.05$, ns denotes not significant. Bars represent mean +/- SEM with individual data points plotted as symbols. **(D)** Images of ring glands from *P0206>UAS-GFP* or *spok>UAS-GFP* larvae reared in normoxia or 5% oxygen from 120 hAEL and dissected at 144 hAEL.
