# Supplementary material for: Hypoxia delays steroid-induced developmental maturation in Drosophila by suppressing EGF signaling
Source: PLoS Genet. 2024 Apr 26;20(4):e1011232. doi: 10.1371/journal.pgen.1011232 (PMC11098494; doi:10.1371/journal.pgen.1011232)
Supplement: S4 Fig — (A) Relative mRNA levels of HIF-1α target gene, fatiga from whole-larvae qRT-PCR of larvae, either da>+ or da>sima-i, reared in ambient oxygen or in 5% O2 from 24 h. n (# of independent samples) ≥ 3 per condition. (B) Pupal volume of w1118 larvae either raised at ambient oxygen (‘N’) or 5% oxygen from 120 h AEL (‘H’). n (# of pupae) = 102 (N, phm > UAS-Dicer),100 (N, phm>UAS-Dicer, sima-i),60 (H, phm > UAS-Dicer), 66 (H, phm>UAS-Dicer, sima-i) (C) Mean time to pupation of larvae, either elav>+ or elav>sima-i, reared in either normal oxygen conditions throughout development (‘N’) or shifted to 5% O2 at 120 h AEL (‘H’). n (# of vials of 30 larvae) ≥ 3 per condition. * denotes p < 0.05, N.S. denotes not significant. Bars represent mean +/SEM with individual data points plotted as symbols. (PDF) [file pgen.1011232.s004.pdf]

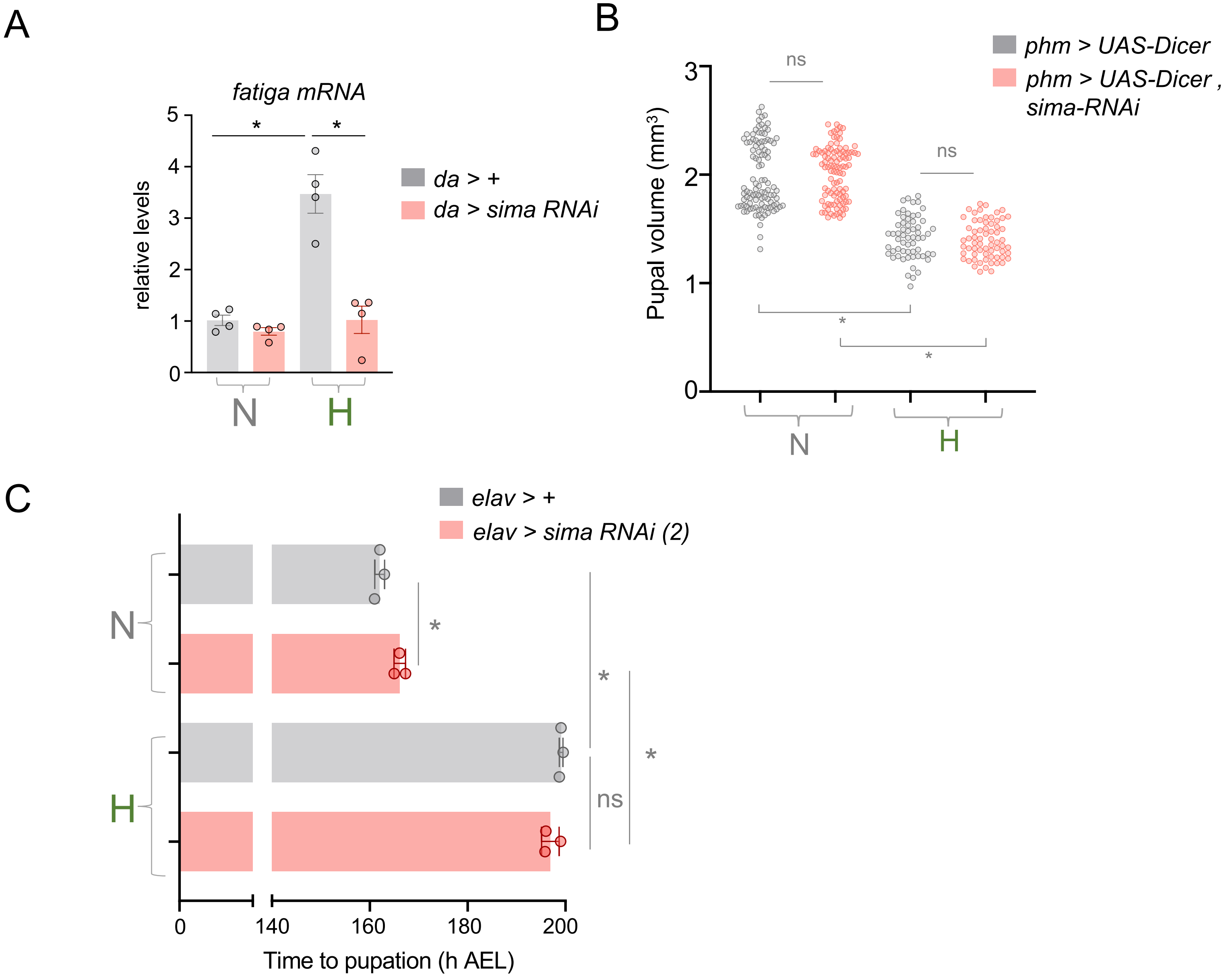

Figure S4 (related to Figure 3). (A) Relative mRNA levels of HIF-1α target gene, *fatiga* from whole-larvae qRT-PCR of larvae, either *da>+* or *da>sima RNAi*, reared in ambient oxygen or in 5% O_2 from 24 h. n (# of independent samples) ≥ 3 per condition. **(B)** Pupal volume of *w[1118] larvae* either raised at ambient oxygen ('N') or 5% oxygen from 120 h AEL ('H'). n (# of pupae) = 102 (N, *phm > UAS-Dicer*),100 (N, *phm>UAS-Dicer, sima RNAi*),60 (H, *phm > UAS-Dicer*), 66 (H, *phm>UAS-Dicer, sima RNAi*) **(C)** Mean time to pupation of larvae, either *elav>+* or *elav>sima RNAi*, reared in either normal oxygen conditions throughout development ('N') or shifted to 5% O_2 at 120 h AEL ('H'). n (# of vials of 30 larvae) ≥ 3 per condition. * denotes p < 0.05, N.S. denotes not significant. Bars represent mean +/- SEM with individual data points plotted as symbols.
