# Supplementary material for: Hypoxia delays steroid-induced developmental maturation in Drosophila by suppressing EGF signaling
Source: PLoS Genet. 2024 Apr 26;20(4):e1011232. doi: 10.1371/journal.pgen.1011232 (PMC11098494; doi:10.1371/journal.pgen.1011232)
Supplement: S5 Fig — (A) Representative images of dilp8-GFP expression in wing discs from 144h AEL larvae reared in either normal oxygen conditions throughout development (‘N’) or shifted to 5% O2 at 120 h AEL. Scale bar indicates 50mm. (B) Relative mRNA levels of dilp8 mRNA, from whole-larvae qRT-PCR of larvae reared in ambient oxygen, 5% O2 from 24 h AEL or 5% O2 from 120 h AEL. Bars represent mean +/SEM with individual data points plotted as symbols. ns denotes not significant. (C) Mean time to pupation of larvae, either da>+ or da>dilp8iRNAi (2), reared in either normal oxygen conditions throughout development (‘N’) or shifted to 5% O2 at 120 h AEL (‘H’). n (# of vials of 30 larvae) ≥ 3 per condition. * denotes p < 0.05, ns denotes not significant. (PDF) [file pgen.1011232.s005.pdf]

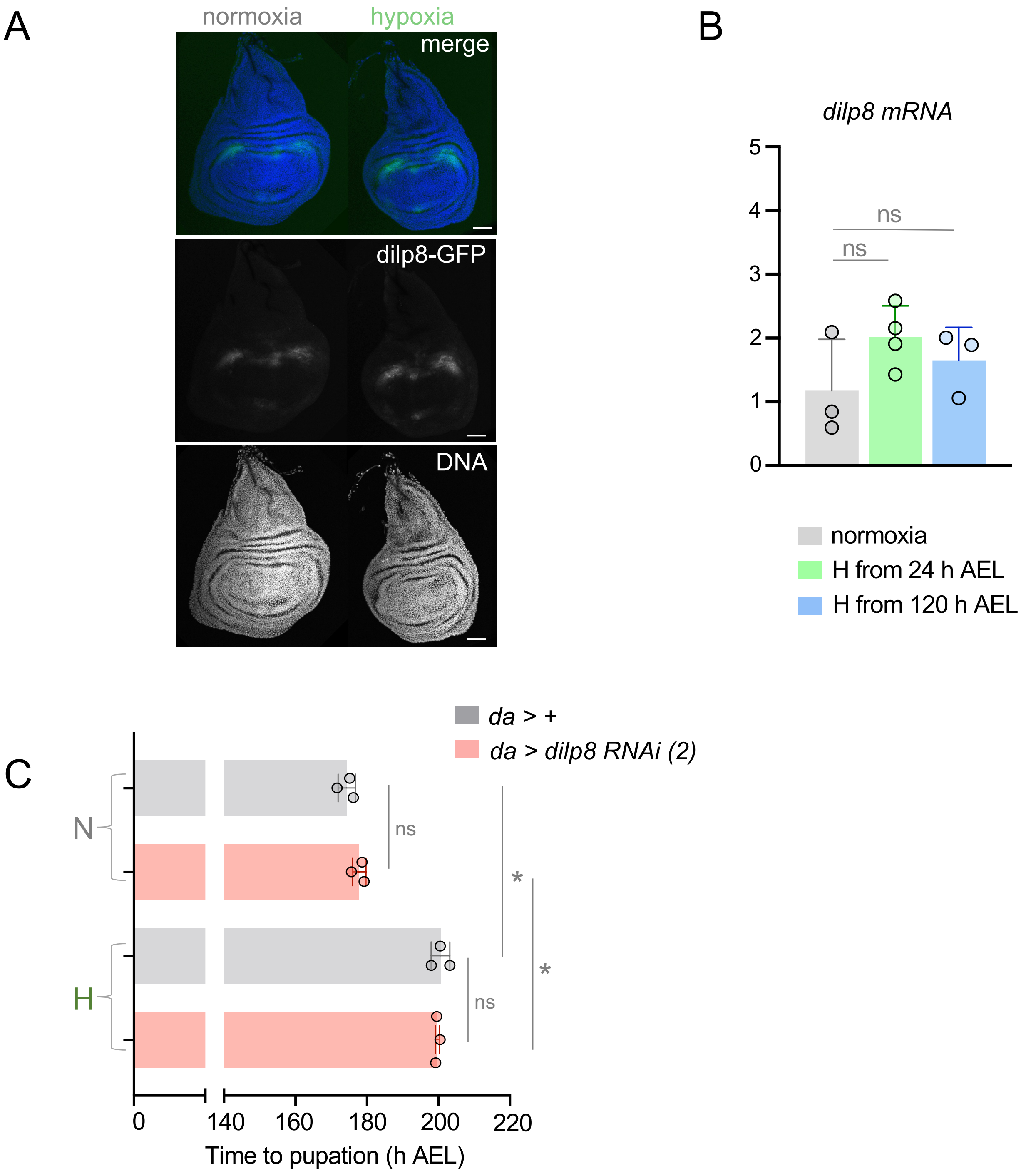

Figure S5 (related to Figure 4). (A) Representative images of dilp8-GFP expression in wing discs from 144h AEL larvae reared in either normal oxygen conditions throughout development ('N') or shifted to 5% O_2 at 120 h AEL. Scale bar indicates 50μm. **(B)** Relative mRNA levels of *dilp8* mRNA, from whole-larvae qRT-PCR of larvae reared in ambient oxygen, 5% O_2 from 24 h AEL or 5% O_2 from 120 h AEL. Bars represent mean +/- SEM with individual data points plotted as symbols. ns denotes not significant. **(C)** Mean time to pupation of larvae, either *da>+* or *da>dilp8iRNAi (2)*, reared in either normal oxygen conditions throughout development ('N') or shifted to 5% O_2 at 120 h AEL ('H'). n (# of vials of 30 larvae) ≥ 3 per condition. * denotes $p < 0.05$, ns denotes not significant.
