# Supplementary material for: Hypoxia delays steroid-induced developmental maturation in Drosophila by suppressing EGF signaling
Source: PLoS Genet. 2024 Apr 26;20(4):e1011232. doi: 10.1371/journal.pgen.1011232 (PMC11098494; doi:10.1371/journal.pgen.1011232)
Supplement: S6 Fig — (A) Average time to pupation of larvae, either w1118 or ptth120F2A (an alternative ptth null mutant) reared in either normal oxygen conditions throughout development (‘N’) or shifted to 5% O2 at 120 h AEL (‘H’). n (# of vials of 30 larvae) ≥ 3 per condition. Bars represent mean +/SEM with individual data points plotted as symbols. * denotes p < 0.05; ns denotes non–significant. (B) Pupal size of animals, either w1118 or mutant for ptth, reared in normoxia or hypoxia from 120 h AEL. Each data point represents body size measured for one animal. n (# of pupae) = 150 (N w1118), 150 (N ptth120F2A), 29 (H w1118), 67 (H ptth120F2A). * denotes p < 0.05. (PDF) [file pgen.1011232.s006.pdf]

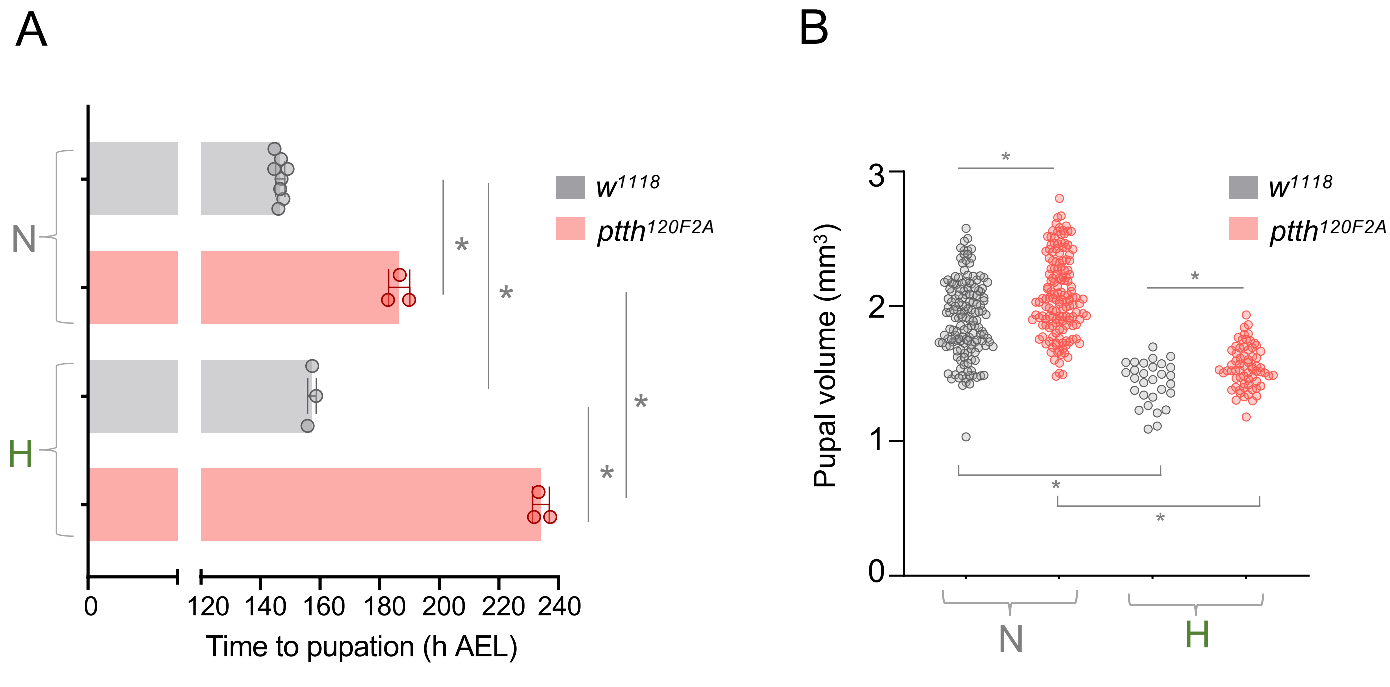

Figure S6. (related to Figure 4). (A) Average time to pupation of larvae, either *w1118* or *ptth120F2A* (*ptth* null mutant) reared in either normal oxygen conditions throughout development ('N') or shifted to 5% O_2 at 120 h AEL ('H'). n (# of vials of 30 larvae) ≥ 3 per condition. Bars represent mean +/- SEM with individual data points plotted as symbols. * denotes $p < 0.05$; ns denotes not significant. **(B)** Pupal size of animals, either *w1118* or mutant for *ptth*, reared in normoxia or hypoxia from 120 h AEL. Each data point represents body size measured for one animal. n (# of pupae) = 150 (N *w1118*), 150 (N *ptth120F2A*), 29 (H *w1118*), 67 (H *ptth120F2A*). * denotes $p < 0.05$.
