# Supplementary material for: Hypoxia delays steroid-induced developmental maturation in Drosophila by suppressing EGF signaling
Source: PLoS Genet. 2024 Apr 26;20(4):e1011232. doi: 10.1371/journal.pgen.1011232 (PMC11098494; doi:10.1371/journal.pgen.1011232)
Supplement: S7 Fig — (A) Average time to pupation of larvae, either spok > + or spok > RafGOF, reared in either normal oxygen conditions throughout development (‘N’) or shifted to 5% O2 at 120 h AEL (‘H’). n (# of vials of 30 larvae) ≥ 3 per condition. (B) Representative images of animals, either phm > + or phm > Egfr-RNAi¸ reared in normoxia throughout development or shifted to 5% O2 at 120 h AEL and imaged at the indicated larval age. In both treatments, phm > Egfr-RNAi arrests development prior to pupation. Scale bar = 1 mm. (C, D) Average time to pupation of larvae expressing RNAi against receptor tyrosine kinases Alk (C) or Pvr (D) under the control of P0206-GAL4 reared in normal oxygen conditions. n (# of vials of 30 larvae) ≥ 3 per condition. (E, F) Relative mRNA levels of Pvr ligands Pvf2 and Pvf3 (E) and Egf ligand vein (F), from whole-larvae qRT-PCR of larvae reared in ambient oxygen, 5% O2 from 24 h AEL or 5% O2 from 120 h AEL. n (# of independent samples) ≥ 3 per condition. Bars represent mean +/SEM with individual data points plotted as symbols. * denotes p < 0.05; ns denotes non–significant. (PDF) [file pgen.1011232.s007.pdf]

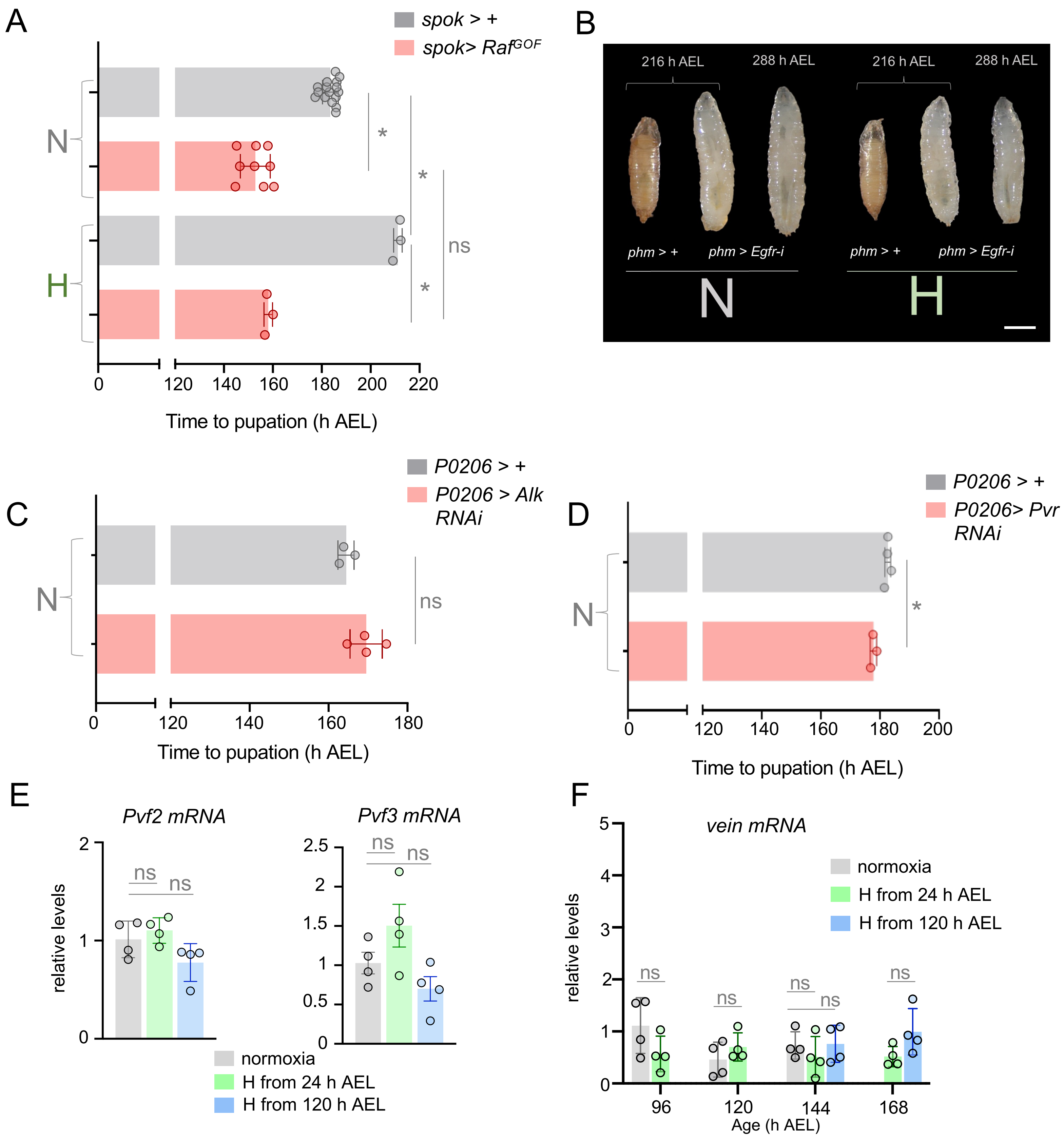

Figure S7. (related to Figures 5 and 6). (A) Average time to pupation of larvae, either *spok* > + or *spok > Raf^GOF*, reared in either normal oxygen conditions throughout development ('N') or shifted to 5% O_2 at 120 h AEL ('H'). n (# of vials of 30 larvae) ≥ 3 per condition. **(B)** Representative images of animals, either *phm* > + or *phm > Egfr-RNAi*, reared in normoxia throughout development or shifted to 5% O_2 at 120 h AEL and imaged at the indicated larval age. In both treatments, *phm > Egfr-RNAi* arrests development prior to pupation. Scale bar = 1 mm. **(C, D)** Average time to pupation of larvae expressing RNAi against receptor tyrosine kinases *Alk* (C) or *Pvr* (D) under the control of *P0206-GAL4* reared in normal oxygen conditions. n (# of vials of 30 larvae) ≥ 3 per condition. **(E, F)** Relative mRNA levels of Pvr ligands *Pvf2* and *Pvf3* (E) and Egf ligand *vein* (F), from whole-larvae qRT-PCR of larvae reared in ambient oxygen, 5% O_2 from 24 h AEL or 5% O_2 from 120 h AEL. n (# of independent samples) ≥ 3 per condition. Bars represent mean +/- SEM with individual data points plotted as symbols. * denotes $p < 0.05$; ns denotes not significant.
