# Supplementary material for: Hypoxia delays steroid-induced developmental maturation in Drosophila by suppressing EGF signaling
Source: PLoS Genet. 2024 Apr 26;20(4):e1011232. doi: 10.1371/journal.pgen.1011232 (PMC11098494; doi:10.1371/journal.pgen.1011232)
Supplement: S8 Fig — (A-D) Average time to pupation of larvae of the indicated genotype, reared in either normal oxygen conditions throughout development (‘N’) or shifted to 5% O2 at 120 h AEL (‘H’). n (# of vials of 30 larvae) ≥ 3 per condition. esg-GAL4, elav-GAL4, mex-GAL4 and r4-GAL4 were used to drive UAS-spi-RNAi expression in the imaginal discs, neurons, enterocytes and fat body, respectively. (E) Relative mRNA levels of Egf ligand spitz, from fat body qRT-PCR of larvae reared in ambient oxygen, 5% O2 from 24 h AEL or 5% O2 from 120 h AEL. n (# of independent samples) ≥ 3 per condition. Bars represent mean +/SEM with individual data points plotted as symbols. * denotes p < 0.05; ns denotes non–significant. (PDF) [file pgen.1011232.s008.pdf]

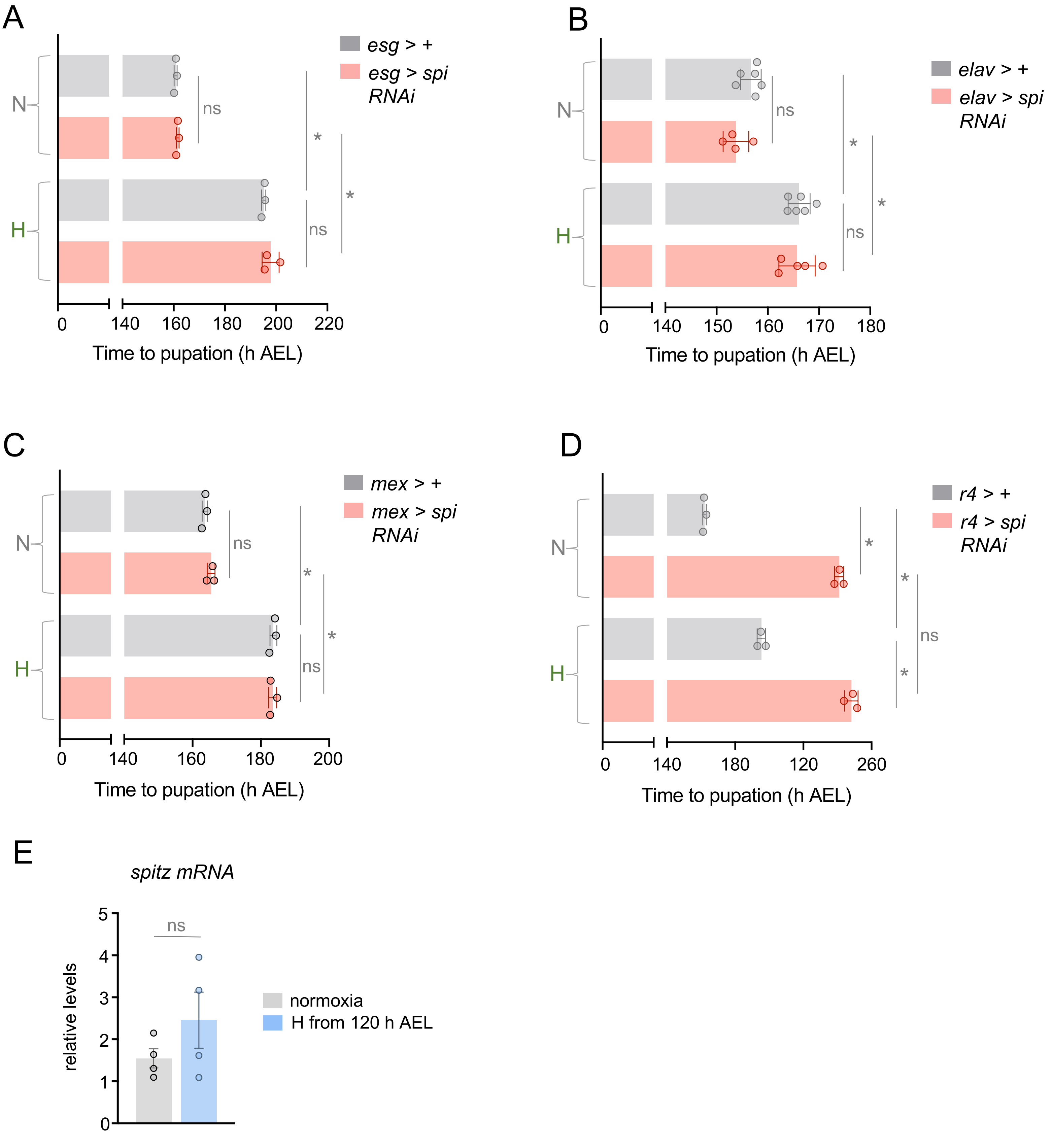

Figure S8. (related to Figure 6). (A-D) Average time to pupation of larvae of the indicated genotype, reared in either normal oxygen conditions throughout development ('N') or shifted to 5% O_2 at 120 h AEL ('H'). n (# of vials of 30 larvae) ≥ 3 per condition. *esg-GAL4*, *elav-GAL4, mex-GAL4 and r4-GAL4* were used to drive *UAS-spi-RNAi* expression in the imaginal discs, neurons, enterocytes and fat body, respectively. **(E)** Relative mRNA levels of EGF ligand *spitz*, from fat body qRT-PCR of larvae reared in ambient oxygen, 5% O_2 from 24 h AEL or 5% O_2 from 120 h AEL. n (# of independent samples) ≥ 3 per condition. Bars represent mean +/- SEM with individual data points plotted as symbols. * denotes $p < 0.05$; ns denotes non -significant.
