# Supplementary material for: Hypoxia delays steroid-induced developmental maturation in Drosophila by suppressing EGF signaling
Source: PLoS Genet. 2024 Apr 26;20(4):e1011232. doi: 10.1371/journal.pgen.1011232 (PMC11098494; doi:10.1371/journal.pgen.1011232)
Supplement: S1 Table — (PDF) [file pgen.1011232.s009.pdf]

Fly Stock	Source
da-GAL4	BDSC #95282
spok-GAL4;UAS-dicer	BDSC #80578
phm-GAL4	BDSC #80577
esg-GAL4	BDSC #84303
elav-GAL4	BDSC #458
mex-GAL4	BDSC #91368
UAS-Egfr-RNAi	BDSC #25781
P[lacW]spi^{s3547}/CyO	BDSC #10462
UAS-sSpi	BDSC #63134
UAS-spi-RNAi	VDRC #3920
UAS-vn-RNAi	VDRC #50358
UAS-Rheb	BDSC #9688
yv TRiP ctrl (II)	BDSC #36304
yv TRiP ctrl (III)	BDSC #36303
w^{1118}	BDSC #5905
GD control line	VDRC #60000
UAS-RafGOF	BDSC #2033
UAS-sima RNAi (#1)	VDRC #106187
UAS-sima-RNAi (TRiP) (#2)	BDSC #33895
KK control line	VDRC #60100
w[*]; TI[RFP[3xP3.cUa]=TI]Ptth[attP]	BDSC #84568
r4-GAL4	BDSC #33832
rn-GAL4	BDSC #78345
UAS-FOXO-RNAi	VDRC #107786
ptth-GAL4	ref 13 McBrayer et al. (2007)
UAS-torso-RNAi	BDSC #33627
P0206-GAL4	Ref 33, Mirth et al 2015
phm-GAL4 (UAS-dicer)	Shimell and O'Connor (2023)
ptth^{120F2A}	ref 12, Shimell et al. (2018)
UAS-λTOP	BDSC #59843
UAS-dilp8-RNAi #1	BDSC #82979
UAS-dilp8 RNAi #2	BDSC #80436
Ilp8:GFP^{MI00727}	BDSC #33079
UAS-mCD8::GFP	BDSC #5137
UAS-NaChBac	BDSC #9469
UAS-Pvr-RNAi	VDRC #13503
UAS-Alk RNAi (TRiP)	BDSC #27518

Table S1: List of fly stocks used in this study
