# Supplementary material for: Hypoxia delays steroid-induced developmental maturation in Drosophila by suppressing EGF signaling
Source: PLoS Genet. 2024 Apr 26;20(4):e1011232. doi: 10.1371/journal.pgen.1011232 (PMC11098494; doi:10.1371/journal.pgen.1011232)
Supplement: S2 Table — (PDF) [file pgen.1011232.s010.pdf]

primer	sequence
fga F	GGAAACGGAAACAGGTCAAA
fga R	TGGTTCATGTCGCTGATGAT
spok F	TATCTCTTGGGCACACTCGCTG
spok R	GCCGAGCTAAATTTCTCCGCTT
phm F	GGATTTCTTTCGGCGCGATGTG
phm R	TGCCTCAGTATCGAAAAGCCGT
vn F	ATGAAGGTGGAGAAGGTGTACAA
vn R	TTGATGGACTTTTCGTCCTTGAA
spi F	CAAATGTCCGGAAACCTTCGAT
spi R	GTCGATCTCCTTGTATTCGCAT
rpl32 F	ATGCTAAGCTGTCGCACAAA
rpl32 R	GTTCGATCCGTAACCGATGT
Br-C F	AGAGCACACCCTGCAAACAC
Br-C R	GCTGCGTGAGTCCAGAGAC
DmHr3 F	GAGGCTTTTCAATCTGAGCATGAA
DmHr3 R	CGATTCCATGTGCAAGATGGAAAT
E75-PB F	TGCAACATCATCCGGAGGAT
E75-PB R	TCCTCCAGATGCAGCATCTCA
pvf2 F	TCAGCGACGAAACGTGCAAGA
pvf2 R	TTTGAATGCGGCGTCGTTCC
pvf3 F	AGCCAAATTTGTGCCGCCAAG
pvf3 R	CTGCGATGCTTACTGCTCTTCACG
dilp8 F	CGACAGAAGGTCCATCGAGT
dilp8 R	GATGCTTGTTGTGCGTTTTG
5S rRNA F	ACGACCATACCACGCTGAAT
5S rRNA R	AGCGGTCCCCCATCTAAGTA

Table S2. List of primers used in this study
