# Supplementary material for: Hypoxia delays steroid-induced developmental maturation in Drosophila by suppressing EGF signaling
Source: PLoS Genet. 2024 Apr 26;20(4):e1011232. doi: 10.1371/journal.pgen.1011232 (PMC11098494; doi:10.1371/journal.pgen.1011232)
Supplement: S3 Table — (PDF) [file pgen.1011232.s011.pdf]

FIGURE	Source of Variation	% of total variation	P value	P value summary
FIG 2F	Interaction	10.6	<0.0001	****
	Hypoxia	80.6	<0.0001	****
	20E feeding	8.871	<0.0001	****
FIG 3A	Interaction	3.746	0.0012	**
	hypoxia	94.58	<0.0001	****
	sima-i	0.4345	0.1322	ns
FIG 3B	Interaction	0.936	0.0042	**
	hypoxia	96.05	<0.0001	****
	sima-i	0.8077	0.0072	**
FIG 3C	Interaction	1.469	0.0375	*
	Hypoxia	96.71	<0.0001	****
	sima-i	0.7186	0.1263	ns
FIG 3D	Interaction	0.6786	0.2523	ns
	hypoxia	93.43	<0.0001	****
	sima-i	0.03499	0.7875	ns
FIG 3E	Interaction	2.505	<0.0001	****
	hypoxia	94.04	<0.0001	****
	sima-i	2.178	0.0001	***
FIG 3F	Interaction	0.03566	0.3804	ns
	hypoxia	97.39	<0.0001	****
	sima-i	0.254	0.0361	*
FIG 4A	Interaction	0.2313	0.3271	ns
	hypoxia	97.97	<0.0001	****
	dilp8-i	0.09831	0.5154	ns
FIG 4B	Interaction	0.1056	0.1455	ns
	hypoxia	92.96	<0.0001	****
	nachbac	0.8311	0.0002	***
FIG 4C	Interaction	1.708	0.0049	**
	hypoxia	52.36	<0.0001	****
	mutant ptth	31.8	<0.0001	****
FIG 4D	Interaction	0.02709	0.5542	ns
	hypoxia	51.16	<0.0001	****
	mutant ptth	7.153	<0.0001	****
FIG 4E	Interaction	3.799	<0.0001	****
	hypoxia	59.22	<0.0001	****
	torso-i	36.66	<0.0001	****
FIG 4F	Interaction	0.008534	0.7716	ns
	hypoxia	29.43	<0.0001	****
	torso-i	24.53	<0.0001	****
FIG 5A	Interaction	3.952	<0.0001	****
	hypoxia	51.36	<0.0001	****
	rafGOF	46.55	<0.0001	****
FIG 5B	Interaction	2.214	0.0531	ns
	hypoxia	77.76	<0.0001	****
	egfr-i	2.899	0.0286	*
FIG 5C	Interaction	2.813	<0.0001	****
	hypoxia	45.21	<0.0001	****
	egfr-i	4.985	<0.0001	****
FIG 5D	Interaction	3.418	<0.0001	****
	hypoxia	86.27	<0.0001	****
	lambda TOP	10.16	<0.0001	****
FIG 6C	Interaction	0.67	0.1951	ns
	hypoxia	71.71	<0.0001	****
	spitz-i	7.568	0.0003	***
FIG 6D	Interaction	7.893	<0.0001	****
	hypoxia	79.42	<0.0001	****
	sSpi	14.48	<0.0001	****

Table S3. Summary of Two-Way ANOVA results

FIG S2D	Interaction	0.3635	0.0296	*
	hypoxia	67.62	<0.0001	****
	20E	1.568	<0.0001	****
FIG S3A	Interaction	0.02278	0.6119	ns
	Hypoxia	98.6	<0.0001	****
	Rheb	0.5339	0.0257	*
FIG S3B	Interaction	0.1676	0.3317	ns
	hypoxia	61.46	<0.0001	****
	rheb	0.5053	0.0926	ns
FIG S3C	Interaction	0.1096	0.6018	ns
	hypoxia	96.63	<0.0001	****
	foxo-i	0.2876	0.4046	ns
FIG S4B	Interaction	0.1984	0.2499	ns
	hypoxia	54.67	<0.0001	****
	sima-i	0.001271	0.9266	ns
FIG S4C	Interaction	0.8513	0.0018	**
	hypoxia	98.74	<0.0001	****
	sima-i	0.07915	0.2006	ns
FIG S5C	Interaction	0.786	0.1027	ns
	hypoxia	97.06	<0.0001	****
	dilp8-i	0.2971	0.2901	ns
FIG S6A	Interaction	6.711	<0.0001	****
	hypoxia	16.75	<0.0001	****
	ptth120f2a	67.04	<0.0001	****
FIG S6B	Interaction	0.09573	0.4229	ns
	hypoxia	35.02	<0.0001	****
	ptth120f2a	2.889	<0.0001	****
FIG S7A	Interaction	5.487	<0.0001	****
	hypoxia	11.9	<0.0001	****
	rafGOF	77.03	<0.0001	****
FIG S8A	Interaction	0.06918	0.3824	ns
	hypoxia	99.05	<0.0001	****
	spitz-i	0.2362	0.126	ns
FIG S8B	Interaction	1.145	0.2858	ns
	hypoxia	83.16	<0.0001	****
	spitz-i	1.995	0.164	ns
FIG S8C	Interaction	0.298	0.1173	ns
	hypoxia	98.75	<0.0001	****
	spitz-i	0.1817	0.2077	ns
FIG S8D	Interaction	3.474	<0.0001	****
	hypoxia	8.288	<0.0001	****
	spitz-i	87.83	<0.0001	****

Table S3 (Cont). Summary of Two-Way ANOVA results
